# CeCILE - An Artificial Intelligence Based Cell-Detection for the Evaluation of Radiation Effects in Eucaryotic Cells

**DOI:** 10.3389/fonc.2021.688333

**Published:** 2021-06-30

**Authors:** Sarah Rudigkeit, Julian B. Reindl, Nicole Matejka, Rika Ramson, Matthias Sammer, Günther Dollinger, Judith Reindl

**Affiliations:** Institut für Angewandte Physik und Messtechnik, Universität der Bundeswehr München, Neubiberg, Germany

**Keywords:** cell-tracking, deep-learning, radiobiology, lifecycle analysis, phase-contrast microscopy

## Abstract

The fundamental basis in the development of novel radiotherapy methods is *in-vitro* cellular studies. To assess different endpoints of cellular reactions to irradiation like proliferation, cell cycle arrest, and cell death, several assays are used in radiobiological research as standard methods. For example, colony forming assay investigates cell survival and Caspase3/7-Sytox assay cell death. The major limitation of these assays is the analysis at a fixed timepoint after irradiation. Thus, not much is known about the reactions before or after the assay is performed. Additionally, these assays need special treatments, which influence cell behavior and health. In this study, a completely new method is proposed to tackle these challenges: A deep-learning algorithm called CeCILE (**Ce**ll **C**lassification and ***I**n-vitro* **L**ifecycle **E**valuation), which is used to detect and analyze cells on videos obtained from phase-contrast microscopy. With this method, we can observe and analyze the behavior and the health conditions of single cells over several days after treatment, up to a sample size of 100 cells per image frame. To train CeCILE, we built a dataset by labeling cells on microscopic images and assign class labels to each cell, which define the cell states in the cell cycle. After successful training of CeCILE, we irradiated CHO-K1 cells with 4 Gy protons, imaged them for 2 days by a microscope equipped with a live-cell-imaging set-up, and analyzed the videos by CeCILE and by hand. From analysis, we gained information about cell numbers, cell divisions, and cell deaths over time. We could show that similar results were achieved in the first proof of principle compared with colony forming and Caspase3/7-Sytox assays in this experiment. Therefore, CeCILE has the potential to assess the same endpoints as state-of-the-art assays but gives extra information about the evolution of cell numbers, cell state, and cell cycle. Additionally, CeCILE will be extended to track individual cells and their descendants throughout the whole video to follow the behavior of each cell and the progeny after irradiation. This tracking method is capable to put radiobiologic research to the next level to obtain a better understanding of the cellular reactions to radiation.

## Introduction

Radiotherapy forms, together with surgery, chemotherapy, and immunotherapy, the four pillars of cancer treatment. Radiation acts on all traversed tissues, resulting in the promising therapeutic outcome of killing tumor cells as well as in acute and late side effects in healthy tissue. The damaging effects of radiation on biological tissue have already been known since the beginning of the 20^th^ century. Since then, there have been efforts to qualify, quantify and understand these effects as well as the disparate reactions of different cell and tissue types (1). In the last 100 years, accompanied by fast technological developments, assays have been developed that measure and quantify radiation sensitivity on large cell populations *in-vitro* (2). This led to fundamental new knowledge on the cellular response including the discovery of cancer stem cells (3, 4) or deep knowledge on the effect of different types of radiation (5, 6). This basic knowledge has been used to improve cancer therapy (7, 8) and risk assessment for radiation exposure, whether medical, occupational, or in space missions (9). Furthermore, it opens the possibility to develop countermeasures or therapy to radiation injury (10).

The gold standard method established in *in-vitro* analysis of direct radiation response is the colony forming assay (CFA) (11). This assay is used to assess radiosensitivity *in-vitro* and to investigate the effects of agents, which are meant to have an impact on the survival when applied before, during, or after radiation exposure of cells. In this assay, the overall ability of cells to proliferate into colonies is used to define the cellular reaction to radiation (10). Although the ability to form colonies is the main quality of cellular response to radiation exposure concerning the reaction of organs or a whole organism, the detail of individual cellular reactions is of interest to analyze and predict the time course of reaction of healthy and tumor tissues. Thus, the time and type of cell death as well as the kind of cell death, i.e., apoptosis, necrosis, or senescence, combined with cell survival results in a more detailed look in the mechanisms of radiation effects (12).

However, this way of performing radiobiology has several challenges: the first is that for each endpoint and each timepoint which is examined by classical approaches, a certain experiment has to be performed requiring a large number of cells (i.e. ten thousand to millions in total) to get statistically significant results (13, 14). This limits the applicability particularly in modern therapy approaches such as particle minibeam or microbeam research, where only small cell numbers are irradiated (15–19). Second, one assay alone is less meaningful since only one property can be studied with each assay. Therefore, different types of assays must be applied to form a comprehensive picture of radiation response. Thus, cells are used in different experiments and samples with slightly different conditions depending on the type of assay. This adds uncertainties to the results and aggravates comparability. Third, the assays are ended at one selected timepoint. This means that the effect is integrated over a certain time interval in some assays or only a snapshot of the effects can be investigated in others. Thus, the time dynamic is lost. The last challenge is, that most of the assays used or at least their evaluation cannot be performed using living cells. Cells must be killed and treated using chemicals such as fixing, permeabilization, or labeling agents. These agents disturb the chemical structures of the cells and might disguise the real radiation effects by adding treatment effects. Furthermore, almost all assays require washing and transferring of cells resulting in the loss of cells that cannot be used for analysis then. This can distort the results especially for high-LET radiation, where many cells die quickly. Some of these challenges can be overcome by increasing the number of performed experiments and thus being able to add more samples, assays, or timepoints per assay. This increases the complexity and the number of necessary investigations per research question.

To analyze the radiation response on a single-cell basis, well-established assays using single-cell analysis such as comet assay (20), fluorescence microscopy (21), or gene sequencing are available (22). These methods of single-cell analysis are time and resource expensive. The more complex and informative a single-cell analysis method gets the fewer amount of cells can be investigated as e.g. in super-resolution microscopy analysis, where only a few cells can be observed in a reasonable time (17, 23). In recent history with further biological developments, such as the use of siRNA or CRIPS/Cas9 and other emerging technologies, it is possible to measure effects also with a low number of cells or even single cells and to increase the throughput (10). Nevertheless, it is quite difficult to interpret these results correctly and the ability to conclude the cellular radiosensitivity and behavior upon radiation exposure with only a single assay is very limited.

This fact leads to a need for a new analysis method, where cells are kept undisturbed in their physical environment and all reactions on a single-cell level can be quantified over several proliferation cycles in one experiment. Such a method is long-term label-free live-cell microscopy (24). State-of-the-art microscopes provide a variety of techniques for label-free live-cell imaging, including phase-contrast, differential interference contrast, and holography-based methods (25, 26). Using these techniques on living cells acquiring videos provides the possibility for accurate tracking of cells and single-cell reactions to radiation exposure. The major challenge is that a huge amount of data is produced, which must be analyzed in detail by detecting and tracking every single cell. Cell tracking methods for microscopic images are already used in radiobiology, mostly for fluorescent images. Here, methods like thresholding (27), region growth (28), or watershed (29, 30) can be applied to segment interesting structures in these images. Also, Forrester and co-workers (31) propose a method for analyzing cell death on time-lapse videos by fluorescent imaging using fluorescent labeling. But detecting and tracking cells on images derived by label-free microscopy is much more challenging. The contrast of the cells compared with the background is low and the cell shape varies throughout the cell cycle. One option for analysis is the identification and labeling of each cell by hand, which is time-consuming and makes the conduction of reasonable and quantitative meaningful radiation experiments almost impossible. Hence, software packages are tackling the recognition problem like cell profiler (32) or the Fiji plugin iTrack4U (33). However, these programs have great limitations, as cell profiler cannot handle single cells in phase-contrast videos and iTrack4U has an optimization for phase-contrast images but is limited to data with a high edge contrast. To achieve this contrast special imaging conditions are needed, where information about the health status and cell cycle are lost. These limitations exclude the use of these programs as standard recognition tools for phase-contrast images in all kinds of brightness and contrast combinations. In the cell segmentation and tracking challenge (34), it was shown that deep-learning based algorithms outperform conventional image analysis approaches in cell detection in phase-contrast images, regarding performance and speed and are even able to outperform human inspection in complex image classification and object detection tasks (35). We, therefore, decided to build an artificial intelligence algorithm that can detect and classify cells in phase-contrast images to be used as a tool for radiobiologic research.

In this study, we introduce a state-of-the-art deep-learning algorithm to solve the complex image analysis task for label-free live-cell imaging. With this method, a model is trained which can automatically evaluate the lifecycle of cells in live-cell microscopy videos. The algorithm can provide information on, among others, the amount, type, and time of cell death, the cell-cycle duration, possible cell cycle arrest and proliferation rate as well as family trees for every single cell including also temporal information. With this powerful method, single-cell reactions can be perfectly studied and differences between cells of a single population can be identified. We are aware of currently existing limitations of the introduced algorithm, regarding the amount of detectable cells and generalization but we also show its great potential for the future. Nevertheless, we decided to publish this first proof-of-principle to use such an algorithm in radiobiological research, as since decades a method of this kind is urgently needed. We think it is important to address as much beneficiaries as possible, to be able to adapt further developments to the needs of possible users in future.

We propose, in the first step, to use phase-contrast imaging. This technology is the most common contrast-enhancing technology, which is normally included in a well-equipped laboratory microscope. Furthermore, good and reliable videos can be acquired with less amount of data, compared to e.g. holographic methods. Nevertheless, recognizing cells on phase-contrast images is a challenging task, as cells in culture have poor contrast. Additionally, depending on cell cycle phase, cells show different shapes and morphologies. Therefore, simple methods like thresholding or region growth, which rely on the intensity of regions for differentiation to segment the cells, cannot be applied. Rather, a method based on pattern recognition is needed. In the last decade, one method for recognizing patterns on images was most successful – the deep-learning based Convolutional Neural Network (CNN) – and is now used in most of the algorithms for the classification of objects in images (36–38). The accuracy of the CNN is highly dependent on the datasets used for training and validation and the CNN architecture. The model ResNet-101 (39) is best suited for classification due to its high accuracy in a short training time and is commonly used in object detectors (40, 41). Therefore, the algorithm developed in this study takes the ResNet-101 as a basis.

The detection of objects, i.e. the identification of an object within an image containing an unknown number of objects together with the correct classification, is an even more complex task. There are many approaches to solve this, where the most accurate results are achieved by the RCNN (Region based Convolutional Neural Network) family. This model family outperforms other commonly used models like the YOLO (You Only Look Once) family in terms of recognizing tiny objects and detecting them in crowded areas (42), which is the case for cells on microscopic images. All members of the RCNN family are two-stage detectors. Hence, these models consist of two separate networks. One is responsible for detecting objects in an image (learning the so-called “objectness”) and predicting their bounding boxes. The other network classifies these objects with a CNN. The computationally most efficient and most accurate network of this family is the “faster RCNN” (43). Its efficiency is due to the usage of a backbone fully convolutional network which extracts a feature map from the input image from where the predictions can be made. This approach makes the algorithm additionally end-to-end trainable resulting in high accuracy. Furthermore, this model is well established, and fast in training and is the commonly used building block in many object detection tasks (41, 44, 45). Hence, this model is chosen as a basis for CeCILE.

In this study, we introduce the faster RCNN based algorithm CeCILE (**Ce**ll **C**lassification and ***I**n-vitro* **L**ifecycle **E**valuation), which can detect and classify cells of three different categories of vital cells (living, round, and dividing) and one category of dead cells in live-cell phase-contrast videos. We show the whole process of the creation of a proper data set up to the final object detection algorithm. Furthermore, we test the algorithm in a radiobiological experiment, where we irradiated CHO cells *in-vitro* with 4 Gy of 20 MeV protons. The results achieved with the algorithm accompanied by manually analysis, as the performance of CeCILE is limited at the moment, are compared to cell survival measured with the gold-standard colony forming assay as well as FACS (fluorescence activated cell sorting) based apoptosis and necrosis assay.

## Materials and Methods

### Cell Culture

For the experiments in this study, two epithelial cell lines Chinese Ovarian hamster cells (CHO-K1) and human cervical carcinoma cells (HeLa) were used.

CHO-K1 were used for the radiation experiments and the generation of the dataset. The cells were cultivated in RPMI growth medium (R8758-500ML, Sigma Aldrich, USA), supplemented with 10 % FCS (F0804-500ML, Sigma Aldrich, USA), 1 % Penicillin-Streptomycin (P4333-100ML, Sigma Aldrich, USA) and 1 % Sodium Pyruvate (S8636-100ML, Sigma Aldrich, USA) grown at a temperature of 37°C, 5 % CO_2_ and 100 % humidity, which is denoted in the following as cell culture conditions.

Additionally, HeLa cells were used to generate the dataset for training the algorithm. HeLa cells were cultivated in RPMI growth medium (R8758-500ML, Sigma Aldrich, USA) supplemented with 10 % FCS (F0804-500ML, Sigma Aldrich, USA) and 1 % Penicillin-Streptomycin (P4333-100ML, Sigma Aldrich, USA) at cell culture conditions.

### Irradiation

The experiments were performed at the ion-microprobe SNAKE (46, 47) at the tandem accelerator of the Maier-Leibniz-Laboratorium in Garching near Munich, Germany.

#### Irradiation and Sample Preparation for CFA Assay and Caspase 3/7-Sytox Assay

CHO-K1 cells for Colony-forming and Caspase3/7-Sytox assay were seeded 24 h before irradiation in self-designed sample holders (15). These sample holders keep the cells under physiological conditions and saturated atmosphere, while there is no medium on the cells during irradiation. A detailed description can be found in (15, 47). In these containers, the cells grow on a 6 µm Mylar foil coated with gelatin to encourage the growth on the foil. For coating, the gelatin was warmed up to 37°C and solved in distilled water to a 0.1 % (w/w) solution. 1 ml of the solution was then added on the Mylar foil in the area of the sample holders, where the closed sample holder has a window only covered by two Mylar foils, and incubated for 30 min at 37°C. Then the gelatin solution was removed and the sample holder was washed two times with PBS. Finally, the sample holders dried on air for a minimum of 2 h. For seeding the cells in a well-restricted area of approx. 6 mm x 6 mm, a silicon insert (Culture insert 2 well, Ibidi, Germany) was used. This insert restricts the growth area to two rectangular areas with a gap of 500 µm in between. The inserts stick themselves on the gelatin-coated stretched Mylar foil and every insert was put in the middle of the window area of the sample holders at the same position by using a self-made template. In each well of the insert, 30.000 cells were added in 100 µl growth medium. CHO-K1 cells were then incubated in the inserts for 24 h at cell culture conditions. Before irradiation, the insert was removed, 3 ml growth medium was added, and the sample holder was closed. Five samples were irradiated for colony forming assay and four samples for Caspase 3/7-Sytox assay. The field size of the irradiation field was 6.5 mm x 6.5 mm. The CHO-K1 cell samples were mounted in the irradiation position in the beamline at an upright rotated microscope. With this microscope, the position of the cells could be visualized and the sample holders could be aligned to the beam. The irradiation procedure in upright position lasted about 10 min. Consequently, the unirradiated sham samples were treated the same as irradiated samples, without switching on the irradiation. The CHO-K1 Cells were irradiated with a target dose of 4 Gy of 20 MeV protons at a dose-rate of 3.7 Gy/min. The dose was monitored during irradiation with an ionization chamber between the sample and the ion beam. The detector was connected to an electrostatic beam switch, which switched off the beam after the dose limit was reached as given from the ionization chamber. The ionization was calibrated with EBT3 gafchromic films (Ashland Advanced Materials, USA) and verified for each irradiation by a film placed behind the sample. The dosimetric measurements using the EBT3 gafchromic films showed an actual mean irradiation dose of (3.8 ± 0.7) Gy (standard deviation, cf. **Supplementary Tables 3** and **4**). This dose will later be referred as the 4 Gy irradiation. Variation of dose came from variations in ionization gas concentrations and beam current variations coming from the accelerator, which could not be fully compensated. The mean dose was calculated by the 9 irradiated samples. The large dose error originates from an outlier, which received only 2.29 Gy, which almost doubles the standard deviation.

#### Irradiation and Sample Preparation for Phase-Contrast Analysis

CHO-K1 cells were seeded in a self-designed live-cell-imaging (LCI) container as described in detail by Hable and co-workers (48). In this experiment, a glass window was used in the LCI container instead of a scintillator window, because the ion-detection was performed between beam and sample and therefore a scintillator window was no longer needed. To encourage the cells to grow on glass, the glass window was coated with gelatin in the same way as described above, except adding 700 µl of the gelatin solution on the glass window instead of 1 ml, because of the smaller area. A four-well insert (micro insert 4 well, Ibidi, Germany) was used to restrict the growth area. This insert has a circular shape and contains four rectangular wells for growing the cells in smaller restricted rectangular areas. The insert was positioned in the middle of the window of the LCI container. In each well, 1000 cells were added in 10 µl growth medium solution and another 300 µl of medium was added as a medium reservoir on top of the insert. The samples were incubated after seeding for 24 h at cell culture conditions. Before irradiation, the insert was removed and the container was closed. The cells were covered with polypropylene foil to keep them at saturated conditions and prevent drying of the cells during irradiation. For proton irradiation, the LCI container was mounted at the microscope in the beamline and the sample with CHO-K1 cells was aligned to the beam. One of the four wells was irradiated with 4 Gy of 20 MeV protons by moving the sample in a position, where only the cells of one area are irradiated. The second well was left unirradiated and served as sham. The last two wells of cells were irradiated with 4 Gy at different dose-rates and are not analyzed here. After irradiation, the medium was removed and 6 ml medium was added. The irradiated dose was measured using an ionization chamber. The calibration from 33 independent dose measurements, where the measured 4 Gy of the ionization chamber was calibrated against gafchromic films, gave a mean dose of (3.9 ± 0.6) Gy. This dose is in the following referred to as 4 Gy.

#### Irradiation and Sample Preparation for Generation of Data for Training of the Algorithm

HeLa cells were seeded in LCI-containers on a scintillator window. The scintillator window was coated with Cell-TAK (Cat. No. 354240, Corning, USA) to improve cell growth. For coating, 5 µg of Cell-TAK was solved in a 30x-Na-bicarbonate-buffer, added on the surface of the scintillator window, and incubated for 20 min at room temperature. Then, the Cell-TAK solution was removed and the coated surface was washed with sterile water. The samples were then completely dried for a minimum of 2 h. An insert (micro insert 4 well FulTrac, Ibidi, Germany) with small 4 round wells (diameter of 0.4 mm) was placed on the coated surface. In each of the wells, 600 cells were added in 10 µl. The samples were incubated at cell culture conditions for 1 h. Afterward, the insert was gently removed and 6 ml medium was added to the cells and they were incubated for another 23 h at cell culture conditions. For irradiation, the container was closed with polypropylene foil. The sample was positioned in the beamline and aligned for irradiation with the microscope. The issue of underrepresented dead cells in the images was addressed by an additional type of irradiated samples. These were irradiated with 55 MeV carbon ions, which are known to induce a higher amount of cell death compared to 20 MeV protons (49). One cell area on the sample was irradiated with 1 Gy of 55 MeV carbon ions, the second with 2 Gy, and the third area with 4 Gy. The last area was left unirradiated as direct control. The carbon ions were detected with a photomultiplier behind the sample as described before (48).

### Live-Cell Phase-Contrast Imaging

CHO-K1 or HeLa cells were imaged using a standard phase-contrast microscope with a motorized stage (Axio Observer Z1, Zeiss, Germany). Additionally, the microscope was equipped with a stage top incubator (Tokai-hit STX, Tokai-hit, Japan). The cells were kept at culture conditions during the observation. Therefore, we were able to image for more than 5 days. Since the incubator enriches the air within with over 95 % humidity and prevents the sample from drying, the water in the incubator’s water bath has to be refilled every day. Every second day the growth medium in the sample was refilled to ensure optimal conditions for the cell growth. The cells were imaged with a 10x objective (Plan-Apochromat 10x/0.45 Ph1, Zeiss, Germany) and recorded with a camera (AxioCam MRm, Zeiss, Germany) with a pixel size of 6.45 µm x 6.45 µm and a field size of 1388 x 1040 pixels. A 1x adapter (60N-C 1” 1,0x Adapter, Zeiss, Germany) between camera and microscope was used. HeLa cells were imaged with the condenser annuli Ph 2 every 15 min for 5 days. CHO cells were imaged with the condenser annuli Ph 1 every 5 min for 2 days.

### Colony Forming Assay

For colony forming assay, 5 samples were irradiated and 5 samples serve as a sham. Immediately after irradiation, the cells were trypsinized to be removed from the mylar foil and counted two times in a Fuchs-Rosenthal chamber (C-Chip, NanoEntek, South Korea). A total number of 400 to 700 cells was counted for each sample. The cells of each sample were seeded in three 12-well-plates (Greiner, Germany). A seeding density of 100 cells/ml was chosen for unirradiated cells and 400 cells/ml for irradiated cells to ensure similar colony density on the 12-well-plates. In every well of the plates, 1 ml of the cell solution was added. The cells were incubated for 5 days at cell culture conditions in a water-jacketed incubator (Uniequip, Germany). After five days the cells were rinsed with PBS (D8537-500ML, Sigma Aldrich, Germany) and fixed with Methanol (SupraSolv^®^ Methanol, Merck, Germany) for 5 min. They were stained with a 0.1 % crystal violet (Kristallviolett, Merck, Germany) solution for 2 min and finally washed with water and dried at room temperature for one day according to (49). The plates were scanned with GelCount (Oxford Optronix Ltd., UK) and counted manually. For evaluation, only colonies were counted with a minimum of 50 cells. For the analysis, the plating efficiency (PE) is defined as the percentage of cells that have formed a colony of all seeded cells. To calculate the survival fraction (SF) the PE value for a sample was divided by the mean PE value of the unirradiated cells (PE_0_). The mean PE value for the unirradiated cells in this study was 0.53 ± 0.04, where the uncertainty (ΔPE) was the SEM (standard error of the mean) among 5 samples. This measured PE value correlates to the PE of previous experiments (49). The uncertainty of SF (ΔSF) was calculated by using the Gaussian error propagation as

$$\Delta SF=SF\cdot \sqrt{{\left(\frac{\Delta PE}{PE}\right)}^{2}+{\left(\frac{\Delta PE_{0}}{PE_{0}}\right)}^{2}}.$$

The results of the experiment were compared with a reference measurement with 200 kV x-rays (49, 50). These data were fitted by a commonly used linear quadratic curve

$$SF=\;\exp(-(\alpha \cdot D+\beta \cdot D^{2})),$$

where D denotes the irradiated dose and α and β are fitting parameters. For the fit, the parameters α = (0.156 ± 0.045) Gy^-1^ and β = (0.0235 ± 0.0055) Gy^-2^ were derived from the results of two independent experiments, performed by K. Ilicic (49, 50) at comparable conditions as in the experiment described here.

### Caspase3/7-Sytox Assay

Directly after irradiation of the cells, the medium was changed and the cells were incubated for 24 h at cell culture conditions. Then, the cells were trypsinized by keeping the supernatant of all steps. Afterward, cells were stored at room temperature for 45 min while moving them to the analyzing laboratory. Finally, the solution of trypsinized cells and the supernatant were mixed and centrifuged at 500 g for 5 min at room temperature. After centrifugation, the supernatant was removed to the last 1 ml of the fluid, which also contained the cells. This solution was gently vortexed to distribute the cells equally in the fluid. The cells in the solution were stained with Caspase3/7 and Sytox (CellEvent™ Caspase-3/7 Green Flow Cytometry Assay Kit, Invitrogen, USA) by following the instructions of the manufacturer. First 1 µl of CellEvent™ Caspase-3/7 Green Detection Reagent was added to the solution to end up in a final concentration of 500 nM of the reagent and the samples were incubated at 37°C for 30 min. Finally, 1 µl of SYTOX™ AADvanced™ was added to the cell solution to obtain a concentration of 1 µM and the solution was incubated for another 5 min at 37°C. The stained cells were analyzed using the FACSCalibur flow cytometer (BD Biosciences, USA). To detect the Caspase 3/7, a 530/30 bandpass filter (FL1) was used to collect the fluorescence emission after a 488 nm excitation and for Sytox a 690/50 bandpass filter (FL3) was used. The data analysis was performed using CellQuest software (BD Biosciences, USA). Cells with a positive Caspase 3/7 and positive Sytox staining were classified as late apoptotic cells. Cells with a positive Caspase 3/7 and negative Sytox staining were classified as early apoptotic cells. Cells with a negative Caspase 3/7 and positive Sytox staining were denoted as necrotic cells and cells with no staining signal were classified as vital cells.

### Manual Labeling of Cells

For manual labeling, phase-contrast images were used. Images containing several cells were uploaded in the open-source browser-based software VGG image annotator (VIA) (51). Here, cells were labeled using rectangular boxes, and classification, as identified by eye by a human expert, was added as a tag. Identification was based on the development of cell morphology up to 25 frames before and after the labeled frame, corresponding to changes within 4 hours.

### CNN Algorithm

For classification, a CNN with four convolutional layers based on the architecture of LeNet-5 (52) was used. Each of the convolutional layers was followed by a max-pooling layer to minimize the number of learnable parameters. The first convolutional layer applied 32 filters to the images of the dataset, the second convolutional layer had 64 filters and the last two convolutional layers applied 96 filters each. Two fully connected layers with 512 and 4 connections follow the convolutional layers. As activation function ReLU (Rectified Linear Unit) was chosen, which is commonly used in CNNs. ReLU was applied in all convolutional layers and the first fully connected layer. In the second fully connected layer, a softmax function was implemented as activation. The network had a total number of 4,143,236 trainable parameters. This algorithm is python based and was implemented using the TensorFlow 1.12.0 backend. The CNN was trained on the classification dataset. For this dataset the labeled boxes of the dataset of CeCILE were cropped to obtain images containing only one object. To tackle class imbalances, the images of the classes div and round were upsampled via data augmentation methods. In the class round every second image was vertically flipped and for the class div the augmentation methods enhance brightness, contrast, and sharpness, lower brightness and contrast were applied on the images and on vertically flipped images to increase the number of images in this class by a factor of 11. During training time the augmentation methods random rotations, random zoom, random width shift, random horizontal shift and horizontal flip were used for all classes to make the training more robust and to improve the generalization. For training and testing the CNN, the classification dataset was randomly split into 75 % for training and 25 % for testing.

### Faster RCNN Algorithm

The object detector is python based and implemented using the TensorFlow Object Detection API (53) with TensorFlow version 1.12.0. The detailed configuration is described in (54). As a backbone architecture, a ResNet101-CNN was used topped by a classification head and a localization head as designed by Ren et al. (43). The finetuned final parameters for CeCILE are shown in **Table 1** in the **Supplementary**.

Additionally, an algorithm for detecting the cells in a video stream was developed using OpenCV 4 and TensorFlow 1.12.0. The algorithm takes the object proposals from the TensorFlow API and calculates positions and classes of the objects over the video frames. CeCILE was trained on an Nvidia RTX 2080 Super GPU. One training cycle took eight hours. To optimize the algorithm 200 training cycles were performed, each with varying parameters. For training and validation, the dataset was split randomly in subset containing 75 % of the labeled images for training and 25 % for testing.

## Results

### Generation of the Dataset

A dataset containing images with marked locations of the cells and their respective labels is needed for training and validation of the model. We decided to use an easily applicable method to generate the dataset by using rectangular boxes around the cells, so-called bounding boxes. Hence, the dataset can be extended for applications at different cell lines or microscopes. To get a more generalized dataset, CHO-K1 and HeLa cells were used. Both cell lines are epithelial cells, grow in a monolayer and are well established in radiobiology. Furthermore, we imaged the cells with different phase-contrast illuminations, resulting in a variation of brightness, inner cell contrast, and edge contrast. Overall 329 images were manually labeled containing 28,576 cells from 5 different samples with 2 different imaging conditions (imaging modality Ph 2 for HeLa cells and Ph 1 for CHO cells). 46 % of all labeled cells were HeLa cells and the other 53 % were CHO cells. In **Supplementary Figure 1**, example images of both cell lines with their specific imaging modalities are shown. This dataset serves as ground truth for training and validation of CeCILE.

**Figure 1:**
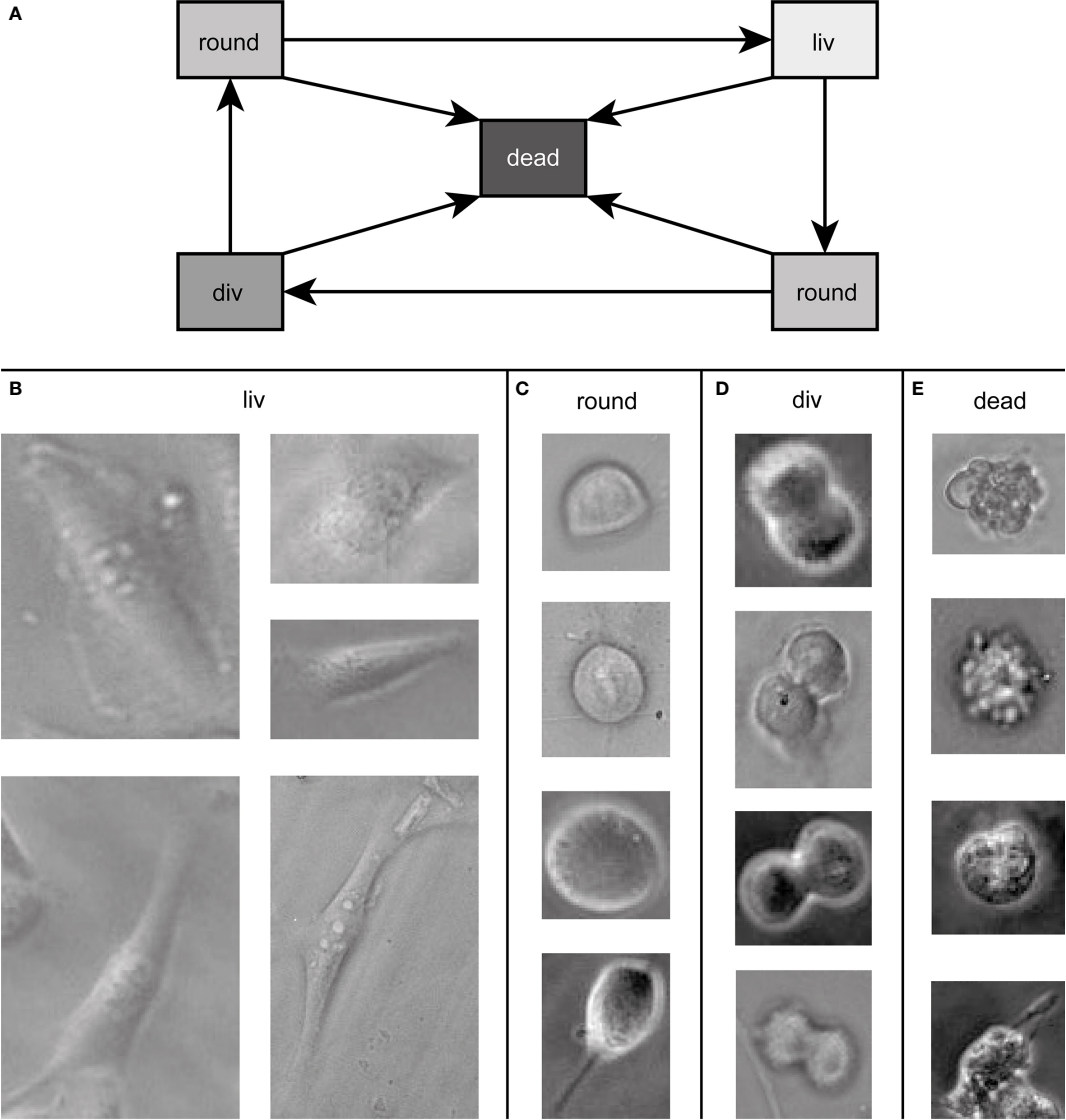
In **(A)** all possible transitions between the classes are depicted. Example images of cells of the dataset in the four states are shown in **(B–E)** defining the classes for training. The contrast of the raw data images was enhanced for better visibility and raw data can be found in the **Supplementary Figure 2**.

Our goal was to follow cells during the whole cell cycle and to be able to detect them in every state. Throughout the cell cycle, the morphology of a cell in culture changes. This change can, in the first approximation, be assigned to three classes. The three classes show a transition circle, which is shown in **Figure 1A**. In the first state, the cell is attached to the surface of the cell culture flask and shows a very flat “fried egg” like shape, where the cell nucleus looks like the yolk and the surrounding part of the cell containing the cell plasma and the organelles looks like the egg white, as shown in **Figure 1B**. Cells of the same cell line can have very different outlines in this stage depending on the environment and mutations. All of them belong to the class living cell (liv). In the second class, the cells are no longer attached to the surface and show a round shape. Therefore, this class is called round cell (round). This state occurs during mitosis shortly before cell division or shortly after division. The round cell has, unlike the living cell, a high contrast at its edges, as shown in **Figure 1C**. Since the transition between living cell and round cell state is smooth, a cell is labeled as round cell when most of the cell edges show this high contrast and therefore, most of the cell is no longer attached to the surface. The third class is named cell division (div) and contains cells that undergo cell division, as shown in **Figure 1D**. A cell is counted as div if it is no longer round, the ongoing separation can be seen and as long as the two daughter cells are not completely separated from each other. These three classes describe vital cells. However, cells can die at any stage of the cell cycle (cf. **Figure 1A**). Therefore, we included a fourth class (dead) in our algorithm, which contains all dead cells. Dead cells typically turn dark inside during the dying process and form bubbles, as shown in **Figure 1E**.

At normal conditions, approx. 80 % of the labeled cells in an image belong to the class liv. This leads to a high imbalance of the dataset. To combat this problem, additional data were acquired, where HeLa cells were irradiated using 55 MeV high-LET carbon ions, increasing the number of dead cells. Overall, the whole dataset including CHO-K1 and HeLa samples contained 65.7 % of the cells in the class liv, 10.6 % in the class round, 0.4 % in the class div, and the last 23.3 % belong to the class dead. All specifications of the dataset are summarized in **Table 2** in the **Supplementary**. An imbalance is still visible in the dataset with an underrepresentation of the classes round, div, and dead. The used algorithms give system-specific methods to compensate for this imbalance and will be explained later on for each case separately.

### CNN Algorithm to Classify Cells

In our first step of developing an algorithm for object detection, we started with a classification task using a simple CNN based on LeNet-5 (52) instead of a more advanced CNN like ResNet-101, because of the much shorter training time. The boxes of the manually labeled images of the dataset were snipped out to get images containing only one object and the algorithm learned to assign class labels to these images. To counteract the imbalance of the dataset, the minor classes were upsampled as described in *Methods* Section *CNN Algorithm* by using the different augmentation methods to obtain 1,320 images in the class div and 4,617 images in the class round forming the classification dataset. In training time additional augmentation was applied on all classes. The augmentation only adds minor changes to the images, which do not affect the appearance of the cells themselves, but for the algorithm, it results in completely new images. A further advantage of augmentation is that these changes make the algorithm more robust and prevent overfitting (55). However, if too many augmentation methods are applied the algorithm tends to overfit and will then not be able to generalize well for analyzing completely new images. Hence, careful evaluation of the classification was performed at each step of training.

**Figure 2:**
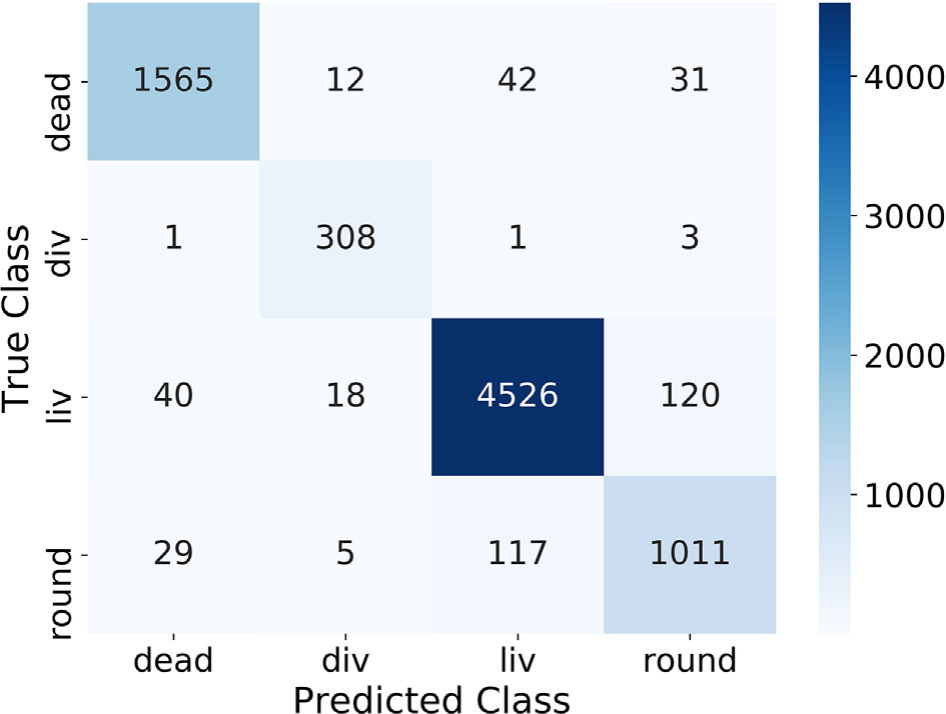
The confusion matrix of the classification of the dataset on a simple CNN.

One important evaluation step is to analyze the confusion matrix on the test dataset (containing 25 % of the classification dataset) depicted in **Figure 2**. The confusion matrix shows the correlation between the manually labeled classes (True Class) of the training dataset and the prediction from the algorithm (Predicted Class) for all cells. The better the algorithm, the more entries are on the main diagonal. **Figure 2** shows that in our case most of the entries are on the main diagonal and therefore most of the images of the test dataset were classified correctly. Overall 5.5 % of the cells were classified in the wrong class. 5.1 % of the dead cells, 1.6 % of the dividing cells, 3.8 % of the living cells, and 13.0 % of the round cells were predicted wrong. The largest error of the algorithm is between the classes round and liv, where 237 cells were predicted wrong. This corresponds to 5.0 % for the class liv and 20.4 % for the class round.

Another evaluation of the algorithm in classification tasks is performed by the scores *precision*, *recall*, and *f*1*_score_* defined as

$$precision=\;\frac{TP}{TP+FP},$$

$$recall=\;\frac{TP}{TP+FN}\text{ and}$$

$$f1_{score}=\;\frac{2\cdot recall\cdot precision}{recall+precision}$$

*TP* indicates the true positives representing the number of cells that are correctly predicted. *FP* are the false positives. These are all images that are falsely predicted to the considered class. The last group are the false negatives (*FN*). They would belong to the considered class but were falsely categorized to another class. These scores must be calculated for each class separately. The *precision* gives the proportion of correctly predicted positive identifications to all positive identifications. It tells how precise are the predictions one gets. *Recall*, on the other hand, is defined by the proportion of actual positives that were correctly identified. This generates the sensitivity of finding positive predictions and how many predictions are missed. The *f*1*_score_* gives the harmonic mean of *precision* and *recall* and therefore provides the quality of the algorithm for each class. All three values are 1.0 for an ideal algorithm.

**Table 1** lists the *precision*, the *recall*, and the *f*1*_score_* of all classes. The *precision* and *recall* scores for all classes lied between 0.87 and 0.98. The classes dead, liv and round have similar *precision* and *recall* scores, while the *recall* score of the class div (0.98) is higher than its *precision* score (0.90). The class liv shows the highest score with an *f*1*_score_* of 0.96 followed by dead and div with *f*1*_score_* of 0.95 and 0.94, whereas the class round has the smallest *f*1*_score_* of 0.87. The mean *f*1*_score_* of all classes is 0.93, which is a very good value and proofs the successful classification using the four classes and acquired dataset.

**Table 1:** Evaluation of the classification by a simple CNN on the upsampled dataset.

	*precision*	*recall*	*f1_score_*
dead	0.96	0.95	0.95
div	0.90	0.98	0.94
liv	0.97	0.96	0.96
round	0.87	0.87	0.87
mean over all classes	0.93	0.94	0.93

### CeCILE - An Algorithm for Cell Detection on Microscopic Videos

After the successful proof of cell classification with the used classes and acquired data, the next step is to set up an object detection. For this study, a detector based on a faster RCNN was designed. The basic idea is to use a backbone fully convolutional neural network to extract the features from an image and to predict the bounding boxes and the classes of the objects in two separate heads. A detailed description of the basic architecture of the faster RCNN can be found elsewhere (43). To save computational time, transfer learning with a pretrained ResNet-101 model trained on the COCO dataset (56) from the TensorFlow 1 Model Detection Zoo was used. Transfer learning provides the basic low level features. For identification of the cell specific appearance, classification, and location, CeCILE was trained on the dataset described in this study. The model was finetuned while training on our dataset with the four classes. The data preparation and training pipeline for faster RCNN is implemented as described by Rosebrock (54) with modifications due to the input data.

**Figure 3:**
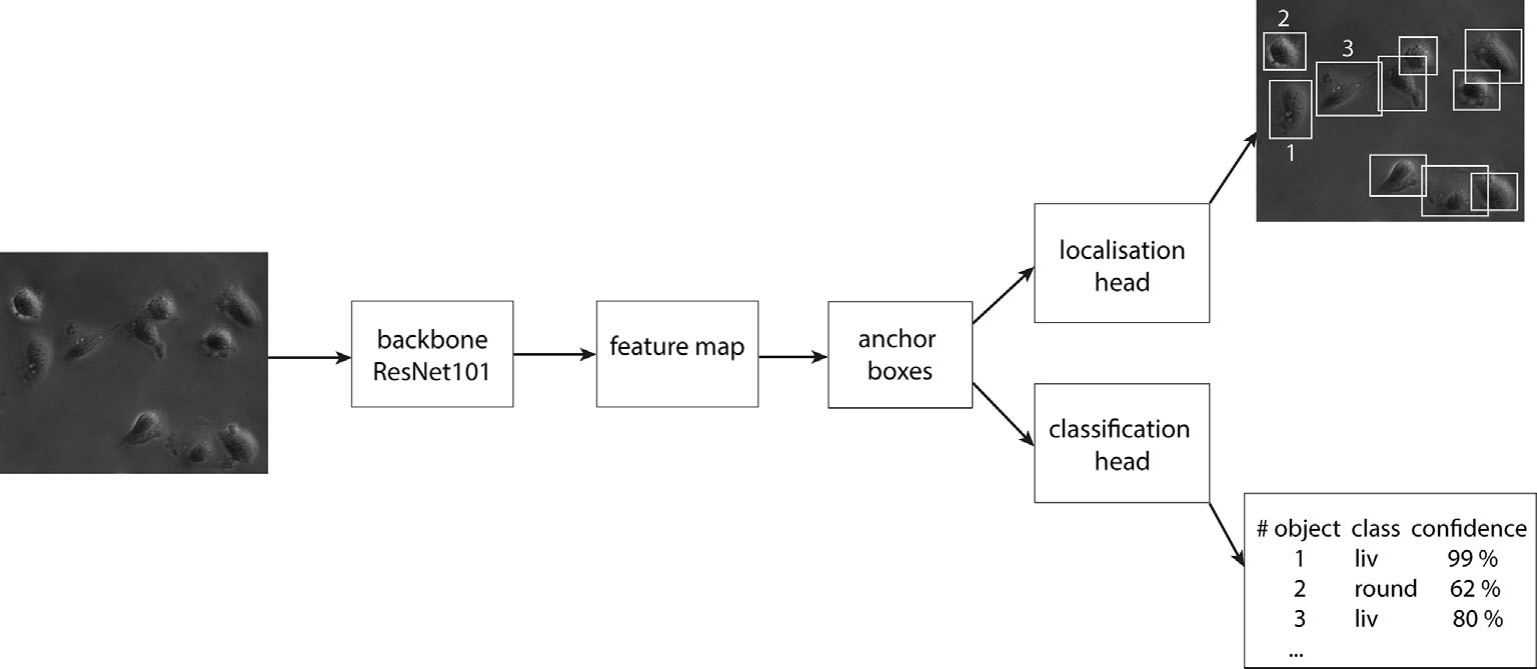
The schematic design of faster RCNN as object detector.

In the first step of the faster RCNN, the image is fed into the backbone convolutional neural network. This gives a representation of image features, the so-called feature map. Afterward, a set of boxes, called anchors, with different aspect ratios and scales is placed around each pixel in the input image. We used aspect ratios of 1:1, 1:2 and 2:1 on four scales of 0.25, 0.5, 1.0 and 2.0. Here, 1.0 equals a size of 256 x 256 pixel. For each anchor, the model infers whether an object is inside or not. The anchor which suits best for each object is finetuned to the position of the object and forwarded as a bounding box. The bounding box defines the object location and extension and is compared for validation to the manually labeled ground truth boxes later. The classification head infers the class label to each bounding box and calculates a confidence score of the algorithm for the respective prediction. In **Figure 3**, a scheme of the design of faster RCNN is displayed. Crowded areas can lead to overlapping bounding boxes of different objects. To account for this, a method called NMS (Non-maximum Suppression) is used, where only bounding boxes are kept with an IoU (Intersection over Union) score smaller than a chosen threshold. The IoU is calculated by dividing the intersection area of two boxes by the union area of the two boxes and is therefore a score for the overlap of two bounding boxes in an image. To increase the generalization of the model, three data augmentation methods were used during training: random image horizontally flips, random brightness, and random contrast adjustments. To compensate for the imbalance of the classes, class weights were implemented. The class liv, containing the majority of the cells, gets the smallest weight of 0.25. The class div gets a four times higher weight and the other two classes both get the medium weight of 0.5. The object detector is trained and optimized for the best classification and detection (cf. **Supplementary Table 1**) in a single image. It was then extended to detect the cells in a video stream forming the final CeCILE algorithm.

### Validation of CeCILE

The performance of CeCILE is qualified using the experimental data of this study. In this study, CHO-K1 cells were in the first sample irradiated with (3.9 ± 0.6) Gy 20 MeV Protons, referred in the following as 4 Gy, and in the second sham irradiated. After irradiation, the cells were imaged via phase-contrast for 2 days every 5 min. Between irradiation and observation, no treatment was applied and the cellular behavior can be investigated without disturbing the cells by any other treatment besides the irradiation. The videos were analyzed by the deep-learning based object detector CeCILE. Additionally, 14 frames in each video were analyzed by a human expert, in the following denoted as ground truth, and the results of both methods were compared. The predictions of CeCILE for both videos are listed in **Supplementary Tables 5** and **6**, from the ground truth in **Supplementary Tables 7** and **8**. The videos with the boxes predicted by CeCILE are shown in **Supplementary Videos 1** and **2**. In order to improve the performance of CeCILE on the videos of the study and to make the image analysis more reliable, 11 frames from the ground truth data from each video were included in the dataset. The frames 288, 432 and 576 were not included here as they contain more than 100 cells. The dataset was split randomly into 75 % for training and 25 % for testing. The generalization of CeCILE was validated on the test data set and on unknown video data.

**Figure 4:**
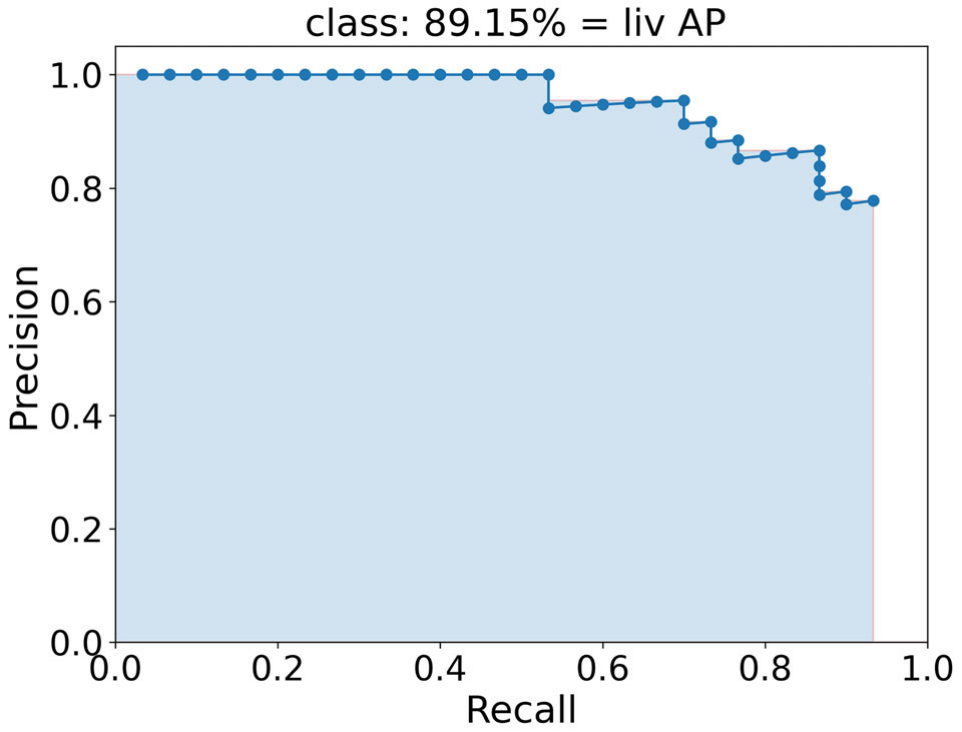
Precision-recall curve (blue dotted line) to calculate the AP of class liv as example. The AP is calculated as the area of the interpolated precision-recall curve colored here in light blue and corresponds to 89.15 %.

To quantify the performance and therefore qualify the algorithm the mAP (mean Average Precision) score, which is commonly used to qualify object detection algorithms (39, 53, 57), is used. In this score, a bounding box is indicated as true positive if the class is predicted right and the predicted box and the ground truth box, i.e. the manually labeled box, overlap by at least a certain percentage defined by the IoU threshold. A bounding box is false positive if either the class is predicted wrong or the box has a smaller overlap with the ground truth box than the IoU threshold. False negative is an object that is not detected at all. The average precision (AP) score is the area under the interpolated precision-recall curve, indicated as light blue area in **Figure 4**. Here, the AP was calculated for the class liv and has a value of 89.15 %. To achieve the precision-recall curve, all boxes of one class are sorted by their confidence score and for each score, the precision and the recall are calculated. Finally, the precision is plotted against the recall and the interpolated area under this curve is calculated as AP. The average AP for each class $\left[\bar{AP}(class)\right]$ is calculated as the mean of each AP at different IoU thresholds from 50 % to 95 % in 10 steps (# *IoU*) of 5 % as

$$\bar{AP}(class)=\;\frac{\Sigma_{IoU=50\%in\;5\%steps}^{95\%}AP(IoU)}{\#\;IoU}$$

This is done for every class separately and the mAP

$$mAP=\frac{\Sigma_{classes(liv,\;round,\;div,\;dead)}\bar{AP}(class)}{\#\;class}$$

is calculated as the mean of all $\bar{AP}(class)$ over all four classes (# *class*). For an ideal algorithm, the mAP equals 100 %.

**Figure 5:**
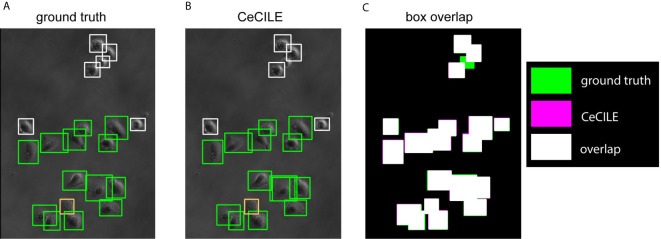
The ground truth boxes are shown in **(A)** and in **(B)** the boxes predicted by CeCILE. White frames label round cells, green frames living cells and orange frames dead cells. In **(C)** the box areas from the ground truth are depicted in green and the box areas of CeCILE in magenta. The overlap of the boxes from CeCILE and the ground truth is shown as white areas.

In **Figures 5A, B** the boxes of the ground truth and the boxes predicted by CeCILE are shown on a phase contrast image for visualization. The overlap of the ground truth boxes and the boxes predicted by CeCILE are depicted in **Figure 5C**. From such an overlap now the mAP-score can be calculated.

**Figure 6:**
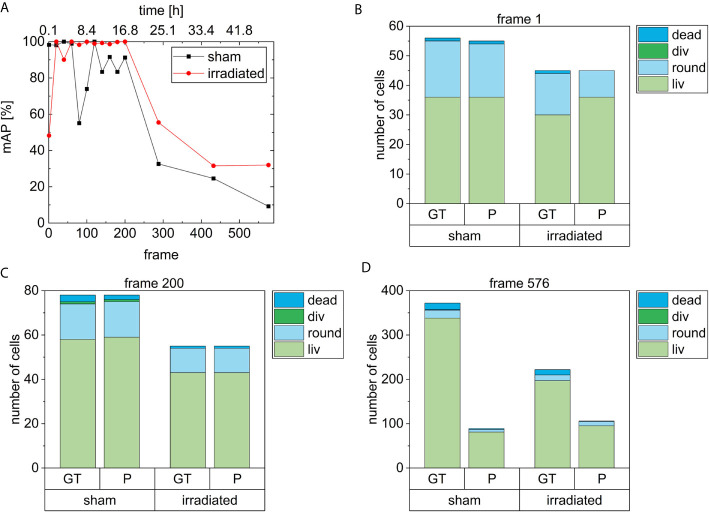
**(A)** shows the mAP scores of the irradiated and the non-irradiated (sham) sample over all frames. In **(B–D)** the number of cells (CHO-K1) in each class are depicted by the bars for frame 1, 200 and 576, respectively. Here, the number of cells of the ground truth labeling (GT) and the prediction of CeCILE (P) are compared.

**Figure 6A** depicts the mAP-score for each analyzed frame of the two samples, one irradiated (red curve) and one sham irradiated control (black curve). The mAP-score in this study was calculated according to (58). For the irradiated sample, the detector gains scores higher than 98 % until frame 200, except from frame 1 (49 %) and frame 40 (90 %). For higher frame numbers, the mAP-score dramatically decreases to scores of 30 % (frame 432) – 60 % (frame 288). In the sham sample, the mAP-scores are higher than 98 % until frame 60. Then, the mAP-score drops to 55 % at frame 80 followed by an increase to 98 % at frame 120. For frames between 140 and 200, the mAP-score is between 83 % and 92 % and decreases after frame 200 quickly to a score of 9 % at frame 576, which corresponds to 48 h after irradiation. The mean mAP-score of the frames of both samples containing less than 100 cells is (91 ± 3) %. The uncertainty is here given by the standard error of the mean. For a better visualization of how the here described mAP-scores were composed, the $\bar{AP}(class)$-scores of the four classes in the here analyzed frames are listed together with the mAP-scores in **Supplementary Table 9**.

In **Figures 6B–D** the number of either predicted cells by CeCILE (P) or manually labeled cells as ground truth (GT) for the four classes are shown in stacked bar graphs. In each graph, the results are shown for one frame. For visualization, frames 1 (**Figure 6B**), 200 (**Figure 6C**) and 576 (**Figure 6D**) are chosen. In the graph for frame 1, the bars in both samples for both the prediction and the ground truth have almost the same height (56 cells and 55 cells in the sham sample and 45 cells in the irradiated sample). For the sham sample, the same number of living cells (36 cells) and of dead cells (1 cell) is determined from both the ground truth and CeCILE, while 1 round cell less is predicted by CeCILE than by the ground truth in this case. On this frame, no cell division is visible. The mAP for this frame in the sham sample is 98 %. In frame 1 of the irradiated sample, CeCILE predicts 6 living cells more than the ground truth and 5 round cells less. The dead cell from the ground truth is not predicted. This discrepancy in classification results in an mAP of 48 %. In frame 200 for both samples, the same cell number is predicted. The cell numbers in the classes round and div coincide between the ground truth and prediction. Whereas, in the class liv, CeCILE predicts 1 cell more than the ground truth and in the class dead CeCILE predicts 1 cell less than the ground truth. This results in an mAP of 91 %. In the irradiated sample, CeCILE predicts the same number of cells for all classes as the ground truth, resulting in an mAP-score of 100 %. At frame 567, CeCILE predicts overall 77 % less cells in the sham sample than the ground truth and 53 % less cells in the irradiated sample. In the sham sample, CeCILE predicts 24 % of the living cells, 33 % of the round cells, and 13 % of the dead cells determined by the ground truth. The cell division is not detected by CeCILE. This results in an mAP-score of 9.23 %. In the irradiated sample, 48 % of the living cells, 77 % of the round cells, and 8 % of the dead cells are predicted by CeCILE compared to the ground truth. The mAP-score is 32 %. The decreased mAP-scores in frame 576 in both samples are not due to the wrong classification as in frame 1 in the irradiated sample, but due to too few detected cells by CeCILE. Therefore, the frames with low mAP-scores can be sorted into these two groups, either with low classification accuracy or low detection efficiency. The decreased performance of CeCILE after frame 200 originates from a problem caused by the algorithm (56), that was used as a basis of the model via transfer learning. In this model, the maximum detections which can be performed on an image are limited to 100 objects. Therefore, our model fails to detect all objects in images with more than 100 objects, which is the case for the frames after frame 200. Solving this problem is currently work in progress.

CeCILE performed very well on partly known video data with frames containing < 100 cells. The generalization of an algorithm provides the performance of the algorithm on unknown data. Here, the generalization is measured on the one hand on the test dataset and on the other hand on unknown video data. CeCILE achieved a mAP of 38.14 % on the test dataset. Since in the test dataset are also images containing more than 100 cells, this might lower generalization than it actually is. Therefore, we retrained CeCILE on our dataset after excluding the ground truth data of the videos of the here described study. Then, the videos were again analyzed by the unknowing CeCILE and the mAP was calculated based on 22 frames of both videos, which are part of the ground truth data. Here, unknowing CeCILE achieved a mAP of 51.51 %. While cells of the class liv and round were predicted reliably with mean $\bar{AP}(class)$-scores of 74.10 % and 70.87 %, the generalization was very low for the classes dead and div with mean $\bar{AP}(class)$-scores of 6.48 % and 16.67 %. The here calculated mAP-scores for each frame are listed in **Supplementary Table 10**.

### Cellular Response to Radiation Evaluated With CeCILE

In the next step, we analyze the cellular response to irradiation with 4 Gy 20 MeV Protons using the phase-contrast videos, which were also used for validation on the section before. For the biological analysis of the videos, the frame number is replaced by the acquisition time with frame 1 being timepoint 5 min and the time between the frames equals 5 min.

**Figure 7:**
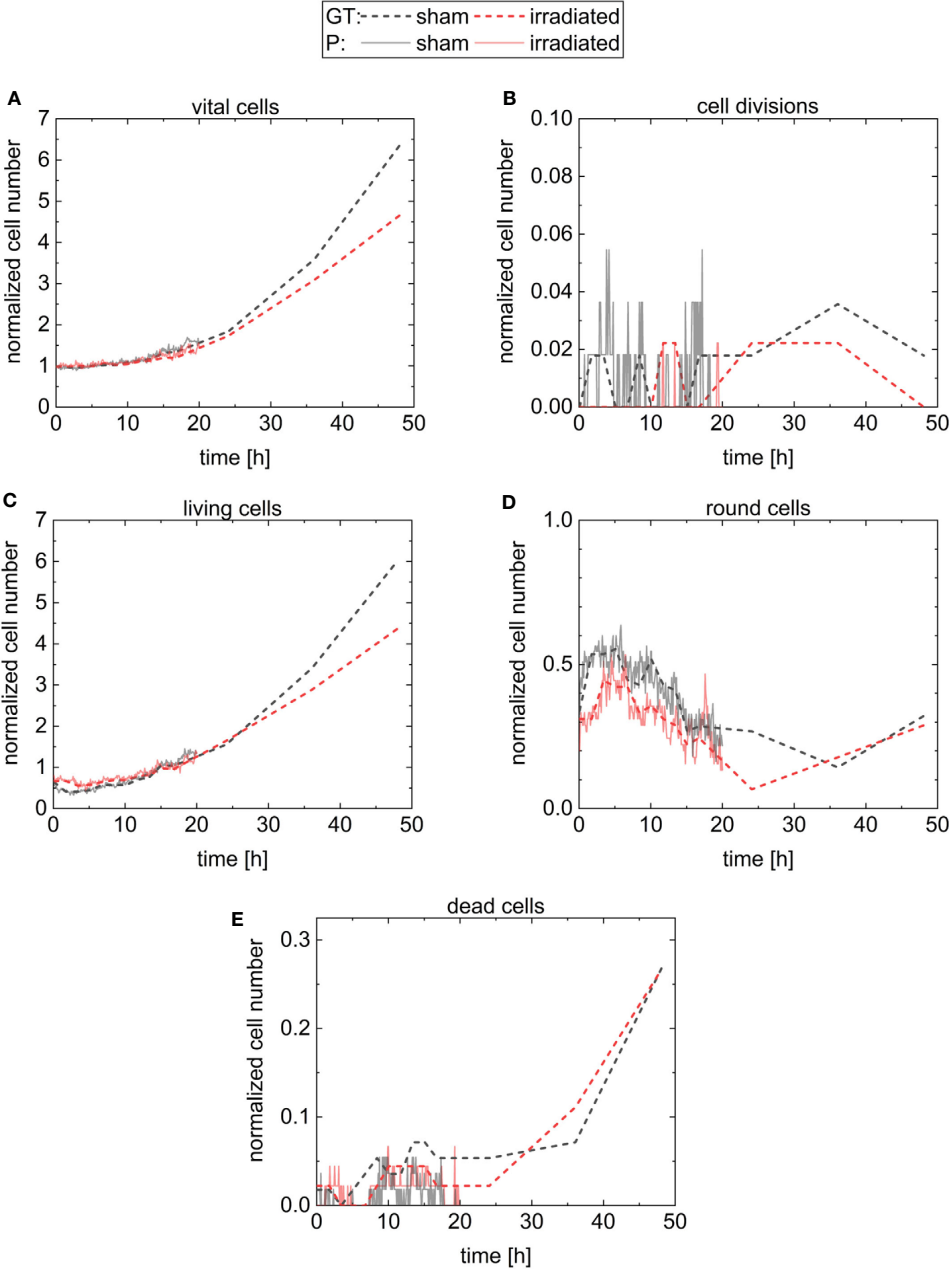
Results for the normalized cell number of the different classes. The number of cells is normalized by the number of cells on the first frame. The classes are vital cells **(A)** containing cells from the three classes cell divisions **(B)**, living cells **(C)**, round cells **(D)**, and the last class is dead cells **(E)**. The results for the sham sample and the irradiated sample are visualized in black and in red, respectively. The predictions of CeCILE (P) are shown by continuous lines for 20 h and the ground truth (GT) by dashed lines for 48 h.

The initial cell number at 5 min is 56 cells (36 liv, 19 round, 0 div, and 1 dead) at the sham sample and 45 cells (30 liv, 14 round, 0 div, and 1 dead) at the irradiated sample. For quantitative analysis, the cell numbers at each timepoint are normalized by the initial cell number in the corresponding sample and are displayed in **Figure 7**. The results of the sham sample are shown in black and in red for the irradiated sample. For comparison, the predictions of CeCILE (P) are shown by continuous lines, while the ground truth (GT) is visualized by dashed lines. In **Figure 7A**, the fraction of all vital cells, i.e. combination of classes liv, div, and round, is shown.

The ground truth shows a phase of constant cell numbers at the beginning of the imaging period up to 5 h. This phase originates from a cell cycle arrest due to the stress of handling during the experiment. At 48 h, the number of cells in the sham sample is 6.4 ± 0.6 times higher compared to the start. A factor of 4.7 ± 0.5 is reached in the irradiated sample, corresponding to 73.4 % growth of irradiated cells compared to sham irradiated controls. These results are statistical different (p < 0.05, unpaired t-test).

For automatic detection using CeCILE in the first 5 h of observation, also here the number of cells stays constant and after that, both populations start to grow with comparable results to the ground truth until 20 h when the normalized number reaches 1.5. For later time points, CeCILE gives no reliable results as already explained above. Therefore, no analysis is done here. In **Figure 7B**, the normalized number of cell divisions is shown. In the irradiated sample, CeCILE detects 6 cell divisions until 20 h with a maximum of 1 div per timepoint and in the sham sample 125 cell divisions until 20 h with a maximum of 3 div per sample. Therefore, cell divisions are detected 21 times more often in the sham sample than in the irradiated sample in the first 20 h. After 20 h, in the ground truth of the irradiated sample no more than 1 cell division per timepoint is observed, whereas, in the sham sample, a maximum of 2 div is detected by the ground truth. The form of the graphs for the living cells (**Figure 7C**) is very similar to the graphs of the vital cells between 10 h and 48 h. Until 3 h, the normalized number of living cells in the sham sample decreases by 36 % from 0.64 to 0.41. After that, it increases steadily to a value of 6.0 at 48 h. The living cells in the irradiated sample decrease by 13 % within the first 5 h from 0.67 to 0.58. From 5 h till the end, it increases steadily to a value of 4.4. Between 3 h and 5 h, where the living cells show their minimum in both groups, the normalized number of round cells in **Figure 7D** increases from 0.32 to a maximum of 0.55 at 5 h in the sham sample and from 0.32 to a maximum of 0.44 at 3.5 h for the irradiated sample. From there, the normalized number of round cells decreases until a minimum is reached for the irradiated cells at 24 h and 37 h for sham cells. Finally, the number of round cells increases to 0.3 at 48 h for both groups. In **Figure 7E**, the normalized number of dead cells is shown. The normalized number of dead cells chitters until 24 h between 0 and 0.044 in the irradiated sample and between 0 and 0.07 in the sham sample. Between 24 h and 48 h, the normalized number of dead cells analyzed by a human expert increases to 0.27 for both groups.

### Cell Survival After Irradiation Using CFA

**Figure 8:**
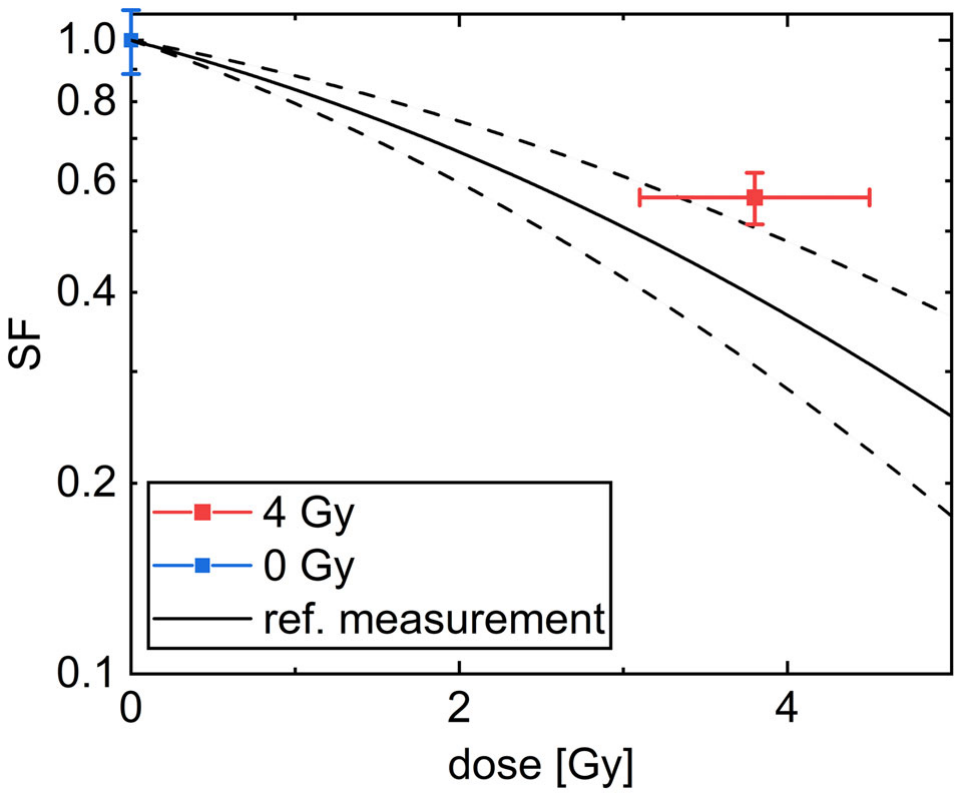
Cell survival curve of CHO-K1 cells obtained after irradiation with 20 MeV protons. The black line denotes a common linear quadratic fit to reference measurements with x-rays with α = (0.156 ± 0.045) 1/Gy and β = (0.0235 ± 0.0055) 1/Gy^2^. The dashed lines indicate the uncertainty range of the fit. The mean over all samples irradiated with a dose of 4 Gy is indicated in red and the mean over the sham samples is indicated in blue. The dose error is the standard error of the mean of the irradiated doses and the SF error is obtained as described in the Methods section.

The colony forming assay is a commonly used assay and is also often referred to as the “gold standard” in radiobiology for addressing cell survival. The cell survival in this assay is determined by the number of cells, which were able to form a colony containing more than 50 cells. In our experiment, the colony forming assay of CHO-K1 cells after irradiation with (3.8 ± 0.7) Gy, referred later as 4 Gy, using 20 MeV protons was used. The results are shown in **Figure 8**.

The SF for the irradiated cells of (56 ± 5) % is significantly smaller than for the unirradiated cells. The results are comparable within the uncertainty range of a reference measurement using x-rays (49, 50), fitted by a common linear quadratic curve, which is indicated by a black line in **Figure 8**. All results are listed in **Supplementary Table 3**. The overlap between the mean SF for the cells irradiated with 4 Gy protons with the reference measurement is decreased when the data point of sample 5, which received only a dose of 2.29 Gy, was excluded. But as this exclusion does not change the meaning of the result we decided to take this data point into account for evaluation.

### Apoptosis and Necrosis After Irradiation Using a Caspase3/7-Sytox Assay

In the second experiment, we measured the number of dead cells and the type of ongoing and completed cell death of irradiated cells. The cells were irradiated with (3.8 ± 0.7) Gy of 20 MeV protons, referred to later as 4 Gy. 24 h after irradiation, all cells were fluorescently stained with a Caspase3/7 and Sytox staining kit. Caspases 3 and 7 indicate if a cell undergoes apoptosis, while Sytox accumulates only in cells with a damaged membrane (59).

**Figure 9:**
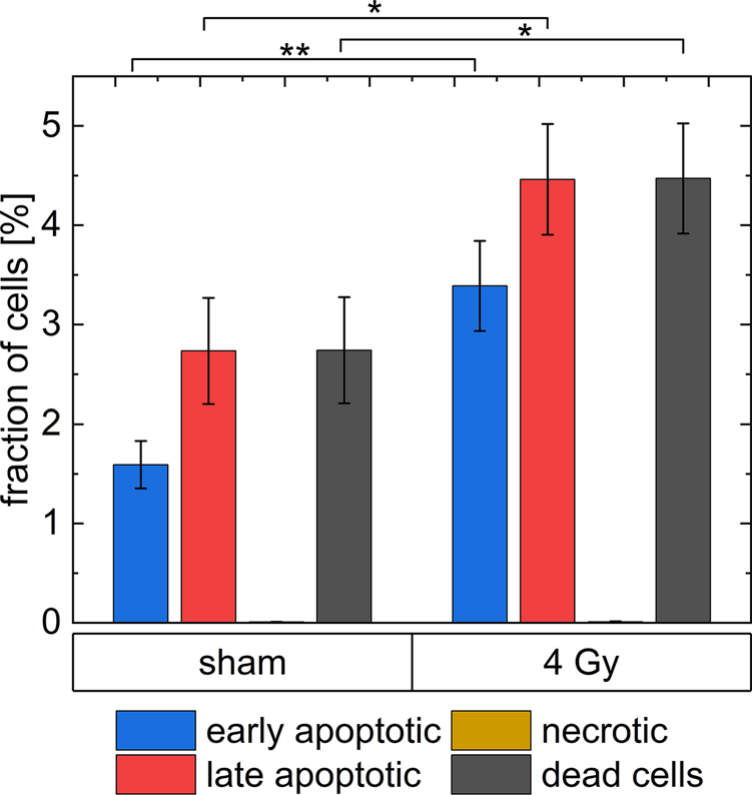
The analysis of dead cells 24 h after irradiation with 4 Gy of 20 MeV protons with a Caspase3/7-Sytox assay. * indicates a p-value < 0.1 and ** a p-value < 0.05. The uncertainties shown as error bars are the standard errors of the respective means.

In **Figure 9**, the results of the Caspase3/7-Sytox assay are shown. The group dead cells includes all cells, which are late apoptotic or necrotic. In the irradiated group (3.4 ± 0.5) % of the cells are in early apoptosis, (4.5 ± 0.6) % of the cells are in late apoptosis and (0.010 ± 0.004) % died due to necrosis. Whereas, (1.59 ± 0.24) % of all cells in the unirradiated group are early apoptotic, (2.7 ± 0.6) % of all cells are late apoptotic and (0.008 ± 0.001) % died due to necrosis. Therefore, 43 % of the apoptotic cells in the irradiated group are early apoptotic and 57 % are late apoptotic, while in the sham samples 37 % of the apoptotic cells are early apoptotic and 63 % are late apoptotic. In the irradiated sample, the percentage of late apoptotic cells is 1.6 times higher than in the sham sample and the percentage of early apoptotic cells is 2 times higher. There is only a small number of necrotic cells (between 0 % and 0.01 %) in the experiment both in irradiated and sham samples. All results are listed in **Supplementary Table 4**.

### Comparison of Clonogenic Survival Between Imaging Analysis and Conventional Methods

Next, we wanted to know, whether the new method based on live-cell phase-contrast imaging gives comparable results to conventional radiobiological assays.

The growth, derived by time lapse imaging from ground truth data follows an exponential function, which is fitted to all data points to model the cell growth. The fit function of the number of normalized cells $n(t)=\frac{N(t)}{N_{0}}$ for t > t_0_ is defined as

$$n(t)=(1-A)+A*\exp\left(\frac{t-t_{0}}{\tau }\right)$$

**Figure 10:**
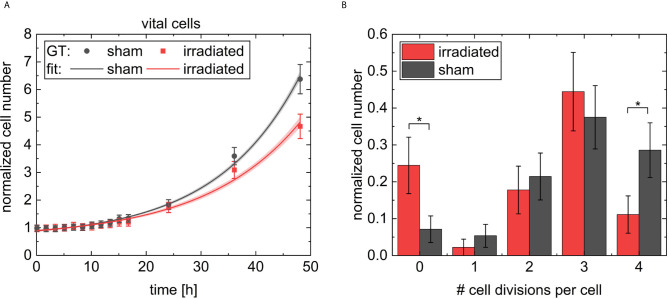
In **(A)** the normalized cell numbers of the vital cells of the ground truth data are shown with dots, the black rectangular dots for the sham sample and red circular dots for the irradiated sample. The data were fitted by exponential growth and indicated as lines. In light red and grey the 63 % confidence band is shown. In B the number of cell divisions per each cell on the two videos followed for 48 h is shown. The results for the irradiated sample are indicated with red bars and for the sham sample with black bars. Errors in **(A, B)** were derived from Poisson statistics. * indicates a p-value < 0.05.

*A* is the amplitude, which is added to the initial number of 1. *t*_0_ is the offset, defined by the cell cycle arrest. *τ* gives the growth constant. The fit parameters for the irradiated sample results are *t*_0_ *_,irr_* = (5.8 ± 1.4) *h*, *A_irr_* = 0.37 ± 0.11 and τ*_irr_* = (17 ± 2) *h* and *t*_0,_ *_sham_* = (5.7 ± 1.0) *h, A_sham_* = 0.37 ± 0.12 and τ*_sham_* = (15.3 ± 0.9) *h* for the sham sample. The goodness of the fit was $X^{2}=0.0008,R_{cor}^{2}=0.97896$, and $X^{2}=0.004,R_{cor}^{2}=0.99239$, respectively. This fit shows different growth constants of (15.3 ± 0.9) h for the faster-growing sham population and (17 ± 2) h for the slowed-down irradiated population. The fits to the data are shown in **Figure 10A**. The cell growth defined cell survival (CGSF) was evaluated at t = 24 h and t = 48 h after 4 Gy irradiation by dividing the corresponding normalized number of vital cells of the irradiated sample by the corresponding normalized number of vital cells of the sham sample resulting in CGSF_24h_ = (91 ± 4) % and CGSF_48h_ = (74 ± 4) %, respectively, where the uncertainties are derived from the 63 % confidence band.

Since the evaluation of the videos are limited due to the fact that CHO cells can grow so densely that they are hard to differentiate and identify, what was the case after 48 h in the sham sample, a longer evaluation of the cells was not possible. However, it was checked whether CGSF values extrapolated to 5 days can serve as a substitute to cell survival obtained from colony formation in future. We, therefore, quantified the maximum number of occurred cell divisions per cell, shown in **Figure 10B**. For example, four cell divisions per cell means that at least one daughter cell is from the fifth generation. Analysis shows significant higher numbers of non-dividing cells in the irradiated sample (p < 0.05) compared to sham sample. Whereas, the majority of cells undergo several divisions in both cases with a maximum of four divisions. The sham sample shows significant higher numbers of cells with four cell divisions compared to the irradiated sample (p < 0.05). CHO-K1 cells show a cell cycle duration of 12 h - 16 h, which would give three to four divisions in 48 h. This is also reflected by the data, measured here.

Colony forming assay shows a cell survival of (56 ± 5) % for 4 Gy 20 MeV proton irradiation. The cells were harvested 5 days after irradiation. When using the derived exponential growth curves an expected CGSF at 5 days of (40 ± 22) % can be estimated originating from different speeds of the irradiated and the sham sample in cellular growth and the cell death occurring in the first days after irradiation. Within the error bars, these two values coincide. Furthermore, it also matches the error band derived in the dose-response curve of the reference measurement with x-rays, which shows an SF of (37 ± 10) % at 4 Gy.

### Comparison of Cell Death Analyzed by CeCILE and a FACS-Based Assay

To be able to compare the data achieved using CeCILE and the FACS data for cell death, a closer look at the FACS analysis working principle is necessary. FACS analysis was performed at 24 h after irradiation, but all cells were counted. Especially the medium was also analyzed to keep all cells that died in the last 24 h for the analysis. For comparison, it is, therefore, necessary to collect all dead cells in the phase-contrast video as well as to track every dead cell, and count the total number of dead cells in the first 24 h. The tracking was done by hand from the images labeled by the algorithm. Dead cells were followed throughout the video until 24 h to make sure that every dead cell is only counted once. In the sham irradiated control, 5 cells died in the first 24 h resulting in a fraction of (4.6 ± 2.1) % (5 out of 108), whereas in the irradiated sample (6.0 ± 2.4) % (5 out of 83) of the cells died. The uncertainties derived from Poisson statistics are, due to the small numbers of dead cells, very high (46 % and 40 %). The differences obtained by CeCILE are, therefore, not statistically significant. Nevertheless, the dead cell fractions are similar but in both cases slightly larger than the results obtained from FACS analysis, where in the irradiated group (4.5 ± 0.6) % of the cells were late apoptotic or necrotic and can therefore be considered as dead. In the sham sample, this fraction is (2.7 ± 0.6) %. Both assays show a trend that irradiated samples show more dead cells than sham samples. The image based data are considered to be not statistically different to FACS data, when comparing irradiated and sham samples of both assays. But a significant difference is visible between the fraction of dead cells when the data for non-irradiated sample analyzed using FACS is compared to irradiated sample from both analysis methods (p < 0.1).

## Discussion

The aim of this study was to demonstrate a novel method for investigating the effects of radiation on eukaryotic cells. This novel method is based on observing the cells for several days after irradiation via live-cell phase-contrast microscopy and analyzing the obtained data with an algorithm based on artificial intelligence. The introduced algorithm called CeCILE can detect cells on microscopic videos and classify them into four cell states depending on their morphology. For the first time, an artificial intelligence based algorithm is presented for the analysis of the response of cells to radiation on live-cell phase-contrast videos. In this study we present the whole process of developing such an algorithm.

First, we needed to set up a dataset of labeled cell images, which can be used to train the algorithm. We separated cells into vital and dead and defined three subclasses by differentiating vital cells further into living, round, and dividing cells. We labeled the cells by surrounding each cell with a rectangular bounding box and tagging the box with the label of the cell class. In the dataset images of two different cell lines, CHO-K1 and HeLa, are included to increase the generalizability and to widen the window of possible applications. We started here for a proof of principle study with these two cell lines, which were often used in radiobiology. For a better generalization, the dataset should be extended with more cell lines depending on the application. However, it has to be tested how good CeCILE generalizes on different cell lines. But as our data set can be quickly extended, CeCILE can be quickly adjusted to different needs. We tested whether our dataset was suitable for training a deep-learning based neuronal network using a simple CNN algorithm, which was trained to classify the cells. The quality is measured with the so-called *F1_score_*, which combines the precision and the recall of the algorithm and has a maximum of 1.00. We achieved an *F1_score_* of 0.93 with precision of 0.93 and recall of 0.94. The largest error is made in the class round, where the precision and recall is 0.87. This means that 13 % of the round cells were not classified as such. This problem occurs since there is a fluent transition between living, round and dividing cells. Therefore, it is difficult for the human expert as well as for the algorithm to sort a cell in the right class when it is directly at the transition step. To minimize the labeling error, the human expert takes the time information into account and looks at the cell morphology several frames before and after the labeled frame. The algorithm instead only gets the single frame image for classification. Therefore, a discrepancy between the human expert decision and the classification from the algorithm is expected. Nevertheless, the most occurring false predictions were round cells classified as living cells or the other way around. Since the transition between the two classes is very continuous, some cells can be classified for both classes and the occurring errors between these classes are negligible. The inaccuracy of the other classes is 5 % for dead cells and much below 5 % for cell divisions and living cells.

The results from classification looked promising so we went further and implemented object detection. CeCILE was developed based on a faster RCNN algorithm, which was headed by a classification and localization head. With this algorithm, it should be possible to detect, locate, and classify every single cell in each frame of a phase-contrast live-cell video, with sufficient accuracy. We faced two major problems developing the algorithm. The algorithm is only able to detect a certain number of cells. When the number of cells in an image exceeds this limit the additional cells are not detected at all. For our algorithm, we used transfer learning meaning that we used a pretrained model (56) which was already able to detect objects in images. In the basic programming architecture of this model, a maximum number of detectable objects is defined. Therefore, we were not able to use images with more than 100 cells. To solve this problem, the basic architecture of the algorithm must be changed which could not be accomplished up to now due to limited resources. The second problem is that the generalization of CeCILE is not yet sufficient. The generalization and performance is measured with the mAP-score, which compares the ground truth (manually labeled cells) with the detection from CeCILE, regarding detection and classification of the cells as well as location and shape of the bounding box. CeCILE achieves a mAP-score of 38.14 % by evaluation on the test dataset and 51.51 % for the evaluation of the unknowing CeCILE on 22 frames of the ground truth data of the two videos of this study. The generalization measured on the test dataset is smaller, because in the test dataset are also images included containing more than 100 cells, therefore the generalization on video data of this study by unknowing CeCILE is here taken into account. For classes liv and round, here, quite high mean $\bar{AP}(class)$-scores of over 70 % were achieved. But for the classes div and dead the generalization was very low, as the mean $\bar{AP}(class)$-scores were 16.67 % and 6.48 %. This low generalization for the two classes came from the fact, that either dead and dividing cells are much less represented in the images as round and living cells. So, here a further extension of the dataset is necessary, preferably with images containing many dead cells as for example after high-LET irradiation or irradiation with x-rays with doses above 10 Gy and images containing many cell divisions, which can be achieved by synchronization of the cell population. Additionally further finetuning of the model will also improve the generalization. To improve the performance on the videos of this study and, therefore, make the analyzation of the videos more reliable, 11 frames in each video were labeled manually and were included in the dataset used for training and testing CeCILE. By giving the algorithm hints by providing ground data is not a new approach and is also used for One-Shot Video Object Segmentation (60). The generation of ground truth data and the retraining of CeCILE is very time consuming. So, this approach limits the applicability in radiobiology and, therefore, our goal is to increase the generalization to be able to analyze unknown videos with CeCILE in future. However, the actual version of CeCILE contained everything for this first proof of principle study when using partly known images that contain less than 100 cells. In our experiment, this corresponds to approximately the first 20 h of imaging. For later timepoints, we based our analysis on the ground truth labeled by a human expert. In the following versions of CeCILE that are under progress, it is planned to extend the number of detectable cells and increase the generalization to use the algorithm routinely in radiobiological analysis. Nevertheless, this basic version of CeCILE already demonstrated the potential of this new analysis method.

We qualified also the performance of the algorithm using the mAP. As expected at times larger than 20 h, the mAP-score decreases as the larger the cell numbers get the more cells are not detected. We, therefore, decided to trust the algorithm only below 20 h. Here, a mean mAP-score of (91 ± 3) % was achieved overall analyzed frames of both the irradiated and the non-irradiated sham sample, containing less than 100 cells. This is a very good result compared e. g. to best performing algorithm on the VOC 2012 test set reaches a mAP of 85 % (61). We conclude that CeCILE in this early stage of developing is very much suited to detect cells in partly known non-labeled phase-contrast live-cell videos.

The next step of development should contain the extension to the detection of non-limited cell numbers, the improvement of the generalization, and the tracking of cells through the videos. This would give the unique opportunity to follow every single cell over several cell divisions and track all changes individually. From this information, the history of each single cell after the irradiation can be evaluated. Thus, occurring cell deaths could also be combined with the cells’ cell-cycle state. This information tells us more about the cause of cell death than the type of cell death (apoptosis, necrosis, etc.) (62) and is, therefore, an important endpoint in radiobiology. The tracking itself can also improve the classification if combined with a logic. For example, a missed cell division could be assigned accordingly, if a second round cell suddenly occurs in the proximity of another round cell in the video. Therefore, the implementation of a tracking method will improve the results further and will increase the evaluation possibilities a lot.

An important step in the qualification of a new analysis method is the comparison to established methods. Therefore, we contrast the detection with the state-of-the-art methods colony forming assay (CFA) for cell survival and FACS analysis for cell death. The assessment of cell survival is based on different principles for CFA and our method. While in the colony forming assay the number of colonies containing more than 50 cells is evaluated 5 days after irradiation, in the microscopic videos the normalized cell numbers in total were recorded. As the performance of CeCILE is in the moment limited we extended the analysis done by CeCILE with a manually analysis of the microscopic videos to show the full potential of our new approach. In future, we plan to improve CeCILE to achieve a fully automated analysis. The evaluation of the image-based analysis shows that up to four cell divisions occur in the analyzed time. Our assay at the moment only counts the number of cells rather than showing a family history of each cell which would be a perfect surrogate for cell survival. We are aware that therefore at the moment the comparison of our data with CFA data has to be taken with caution. This limited comparability of assays reflecting proliferation in some way such as MTT were already addressed in several studies (63–66). One major result of these studies is that under certain circumstances proliferation assays can be used to address the cell survival, as done by CFA. The two major criteria are, that the cells are in exponential growth phase and that a sufficient number of cell divisions is used in the analysis. Both criteria are fulfilled here, which gives a hint that calculations performed here could be used as a prediction for cell survival in the first order. Nevertheless, we think that the analysis performed here can only be a guide to the potential of the algorithm when used on images with the corresponding length, and also when the tracking of each cell through time is possible. Furthermore, the results by CeCILE show the development of the cell population over time. For example growth stagnation in the first 5 h after irradiation could be detected, which can be connected to the enhanced stress of the cells by the handling during the irradiation treatment. This enhanced stress can also be seen in the increased amount of round cells in both populations at this time. So, CeCILE provides additional information besides cell number. We, therefore, conclude that this proof-of-principle was a success but the implementation of a true cell survival measurement needs much more data which are analyzed quantitatively and with more detail in the future. To do so further improvements on CeCILE are necessary. At the moment the limiting factor in the analysis of the videos is the increasing cell density over time on the sample until the cells could no longer be properly distinguished on the videos. In future experiments, a smaller initial cell density and a larger observation field can lead to a longer recording time and therefore to a better analysis by CeCILE. To further improve the measure of cell survival by CeCILE a tracking method will be implemented, that will measure the proliferation on a single-cell level and will also be able to quantify the cell cycle of each single cell. With this approach also events like cell death can be correlated to the cell cycle and the cellular history and lineage.

By looking at the analysis of cell death within 24 h after irradiation, similar results within the uncertainty range were achieved by the Caspase3/7-Sytox assay and the analysis by CeCILE. While the results of the FACS analysis were statistically different by a p-value of 0.1, the results of CeCILE were not significantly different, because of the high uncertainty coming from Poisson statistics and the low initial cell number. To decrease these uncertainties we plan to increase the observation field on the samples in future experiments. In the next version of CeCILE it is planned to overcome the limitation of detectable cells. Therefore, the numbers of analyzed cells will be dramatically increased, which leads to a significant decrease of the error. Nevertheless, the fact that results of both methods are in the same range, shows the potential of our new method. Another aspect is, that the Caspase3/7-Sytox assay can additionally differ between necrosis, late and early apoptosis. However, differing between different cell deaths doesn’t answer the question of the cause for cell death. So here an approach, which investigates the circumstances of cell death would provide more information for the radiobiologic research (62). Extending CeCILE with a tracking method will allow to analyze this endpoint.

To conclude, we introduced a new analysis method to automatically detect and quantify the radiation response of single cells in live-cell phase-contrast images using the deep-learning based algorithm CeCILE. This algorithm shows great potential for application in radiobiology. It is easy to use and it shows already a high accuracy when compared to manual assignments. CeCILE has the potential to analyze the same endpoints as state-of-the-art assays. Besides, it can give information on cell status, cell cycle duration, and once finalized about the lineage of every single cell. It furthermore exceeds conventional radiobiological methods as cells are observed and analyzed under physiological conditions and additional time information can be gathered. We conclude that this new method enables fast evaluations of phase-contrast microscopic data to gain new and deeper insights in the field of radiobiology.

## Data Availability Statement

The original contributions presented in the study are included in the article/**Supplementary Material**. Further inquiries can be directed to the corresponding author.

## Author Contributions

SR and JR wrote the paper. The experiments were performed by SR, NM, MS, and JR. The data set was generated by RR and SR. The algorithm CeCILE was written and adapted by JBR and SR. GD, SR, JR, and NM discussed the results. All authors contributed to the article and approved the submitted version.

## Funding

Funded by transnational access program RADIATE.

## Conflict of Interest

The authors declare that the research was conducted in the absence of any commercial or financial relationships that could be construed as a potential conflict of interest.
