# Supplementary material for: CeCILE - An Artificial Intelligence Based Cell-Detection for the Evaluation of Radiation Effects in Eucaryotic Cells
Source: Front Oncol. 2021 Jun 30;11:688333. doi: 10.3389/fonc.2021.688333 (PMC8278143; doi:10.3389/fonc.2021.688333)
Supplement: Supplementary file 1 [file DataSheet_1.pdf]

Supplementary

Table 1: The optimal parameter after hyperparameter tuning for CeCILE.

Parameter	
Number of classes	4
Epochs	120000
Anchor aspect ratios	0.5, 1.0 and 2.0
First stage NMS IoU threshold	0.7
First stage maximum proposals	400
First stage localization loss weight	1.0
First stage objectness loss weight	2.0
Dropout keep probability	0.8
Second stage NMS IoU threshold	0.8
Maximum detections per class	400
Maximum total detections	400
Second stage localization loss weight	3.0
Second stage classification loss weight	1.0
Batch size	1
Manual step learning rate	Initial learning rate: 0.003
	Step: 50000 learning rate: 0.0003
Data augmentation methods	Random horizontal flip
	Random adjust contrast
	Random adjust brightness
Class weights	Liv: 0.25
	Round: 0.5
	Dead: 0.5
	Div: 1.0

Table 2: Specifications of the dataset.

Sample number	Cell line	Number of labeled frames per position	Time between labeled frames	Imaging Modality	Imaged positions	Cells per image	Irradiation
1	HeLa	Position 1/2: 70<br>Position 3/4: 42	15 min	Ph 2	4	31 - 107	55 MeV C-ions<br>Position 1: 1 Gy<br>Position 2: 2 Gy<br>Position 3: 4 Gy<br>Position 4: 0 Gy
2	CHO	Position 1/2: 11<br>Position 3/4: 10	1 h 40 min	Ph 1	4	45 - 361	20 MeV protons<br>Position 1: 0 Gy<br>Position 2 - 4:

							4 Gy
3	CHO	Position 1 - 3: 11<br>Position 4: 10	1 h 40 min	Ph 1	4	32 - 347	20 MeV protons<br>Position 1: 0 Gy<br>Position 2 - 4: 4 Gy
4	CHO	7	1 h 40 min	Ph 1	1	57 - 145	No irradiation
5	CHO	6	random	Ph 1	2	72 - 128	No irradiation

Table 3: The results of the colony forming assay of CHO-K1 cells irradiated with 20 MeV protons.

Sample number	Dose	PE	SF
1	4.00	0.293	0.56
2	4.22	0.313	0.59
3	4.18	0.302	0.57
4	3.76	0.327	0.62
5	2.29	0.256	0.49
mean	3.7 ± 0.4	0.298 ± 0.014	0.56 ± 0.05
6	0	0.62	1.18
7	0	0.61	1.15
8	0	0.52	0.98
9	0	0.45	0.85
10	0	0.45	0.84
mean	0	0.53 ± 0.04	1.00 ± 0.12

Table 4: The results of the Caspase3/7-Sytox assay analyzed by a FACS.

Sample	Dose	Caspase positive/ Sytox positive	Caspase negative/ Sytox positive	Caspase positive/ Sytox negative	Caspase negative/ Sytox negative	dead cells (late apoptotic + necrotic)
1	3.72	5.5	0.010	3.1	91.4	5.5
2	4.39	5.1	0.000	3.0	91.9	5.1
3	3.39	2.6	0.020	2.6	94.8	2.6
4	4.40	4.7	0.010	4.9	90.4	4.7
mean	4.00 ± 0.25	4.5 ± 0.6	0.010 ± 0.004	3.4 ± 0.5	92.1 ± 0.8	4.5 ± 0.6
5	0	3.2	0.010	1.07	95.7	3.2
6	0	2.6	0.005	1.97	95.4	2.6
7	0	1.1	0.005	1.17	97.7	1.1
8	0	4.0	0.010	2.16	93.8	4.0
mean	0	2.7 ± 0.6	0.008 ± 0.001	1.59 ± 0.24	95.7 ± 0.7	2.7 ± 0.6

Table 5: Predictions from CeCILE for the video of the 4 Gy irradiated cells. Start X, Start Y, End X, and End Y indicate the coordinates of the boxes in the images.

Table 6: Predictions from CeCILE for the video of the sham irradiated cells. Start X, Start Y, End X, and End Y indicate the coordinates of the boxes in the images.

Table 7: Ground truth predictions for the video of the 4 Gy irradiated cells. Start X, Start Y, End X, and End Y indicate the coordinates of the boxes in the images.

Table 8: Ground truth predictions from the video of the sham irradiated cells. Start X, Start Y, End X, and End Y indicate the coordinates of the boxes in the images.

Table 9: For validation of the performance of CeCILE on the videos of the study, the $\overline{AP}(class)$-scores were calculated for each class by comparing the predictions of CeCILE for the here listed frames on two videos with the ground truth. The mAP is then the mean of the $\overline{AP}(class)$-scores and also listed in this table for each frame. In frames where classes are missing in the ground truth, these classes are indicated with the entry "no object".

frame/video	dead	div	liv	round	mAP
1/sham	100.00	no object	100.00	94.74	98.25
20/sham	100	100	95.65	96.67	98.08
40 /sham	no object	100	100	100	100
60/sham	100	no object	100	96.77	98.92
80/sham	0	no object	83.59	81.67	55.09
100/sham	33.33	100	89.59	72.85	73.94
120/sham	100	no object	100	100	100
140/sham	50	no object	100	100	83.33
160/sham	75	no object	100	99.82	91.61
180/sham	50	no object	100	100	83.33
200/sham	66.67	100	98.25	100	91.23
288/sham	0	0	69.59	60.83	32.61
432/sham	25	0	35.85	37.5	24.59
576/sham	6.67	0	18.04	12.22	9.23
1/irradiated	0	no object	89.15	55.56	48.24
20/irradiated	100	no object	100	100	100
40 /irradiated	no object	no object	90.43	89.74	90.08
60/irradiated	no object	no object	100	100	100
80/irradiated	no object	no object	96.55	100	98.28
100/irradiated	100	no object	99.8	100	99.93
120/irradiated	100	no object	96.77	100	98.92
140/irradiated	100	100	97.14	100	99.29
160/irradiated	100	100	94.52	100	98.63
180/irradiated	100	no object	99.49	100	99.83
200/irradiated	100	no object	100	100	100
288/irradiated	100	0	57.44	64.44	55.47
432/irradiated	0	0	43.2	82.95	31.54
576/irradiated	8.33	no object	39.23	48.33	31.97

Table 10: For measuring the generalization, CeCILE was retrained by excluding the groundtruth data of this study for the dataset CeCILE was trained on. Here, the mean $\overline{AP}(class)$-scores for the four classes are listed for 11 frames of each video

and the mAP-scores of these frames, which the unknowing CeCILE achieved. In frames, where classes are missing in the ground truth, these classes are indicated with the entry "no object".

frame/video	dead	div	liv	round	mAP
1/sham	0.00	no object	78.37	72.38	50.25
20/sham	0.00	0.00	73.19	81.58	38.69
40 /sham	no object	0.00	77.34	78.37	51.90
60/sham	0.00	no object	70.73	67.64	46.12
80/sham	0.00	no object	67.49	68.92	45.47
100/sham	33.33	100.00	86.05	72.73	73.03
120/sham	0.00	no object	79.01	77.84	52.28
140/sham	0.00	no object	89.18	66.11	51.76
160/sham	25.00	no object	70.87	73.67	56.51
180/sham	25.00	no object	78.43	60.00	54.48
200/sham	33.33	0.00	77.24	60.83	42.85
1/irradiated	0.00	no object	78.88	57.14	45.34
20/irradiated	0.00	no object	71.15	60.12	43.76
40 /irradiated	no object	no object	64.05	75.00	69.52
60/irradiated	no object	no object	63.99	77.72	70.86
80/irradiated	no object	no object	68.01	84.21	76.11
100/irradiated	0.00	no object	69.81	85.24	51.68
120/irradiated	0.00	no object	76.02	74.52	50.18
140/irradiated	0.00	0.00	71.75	76.79	37.13
160/irradiated	0.00	0.00	74.08	51.92	31.50
180/irradiated	0.00	no object	68.30	63.75	44.02
200/irradiated	0.00	no object	76.35	72.73	49.69

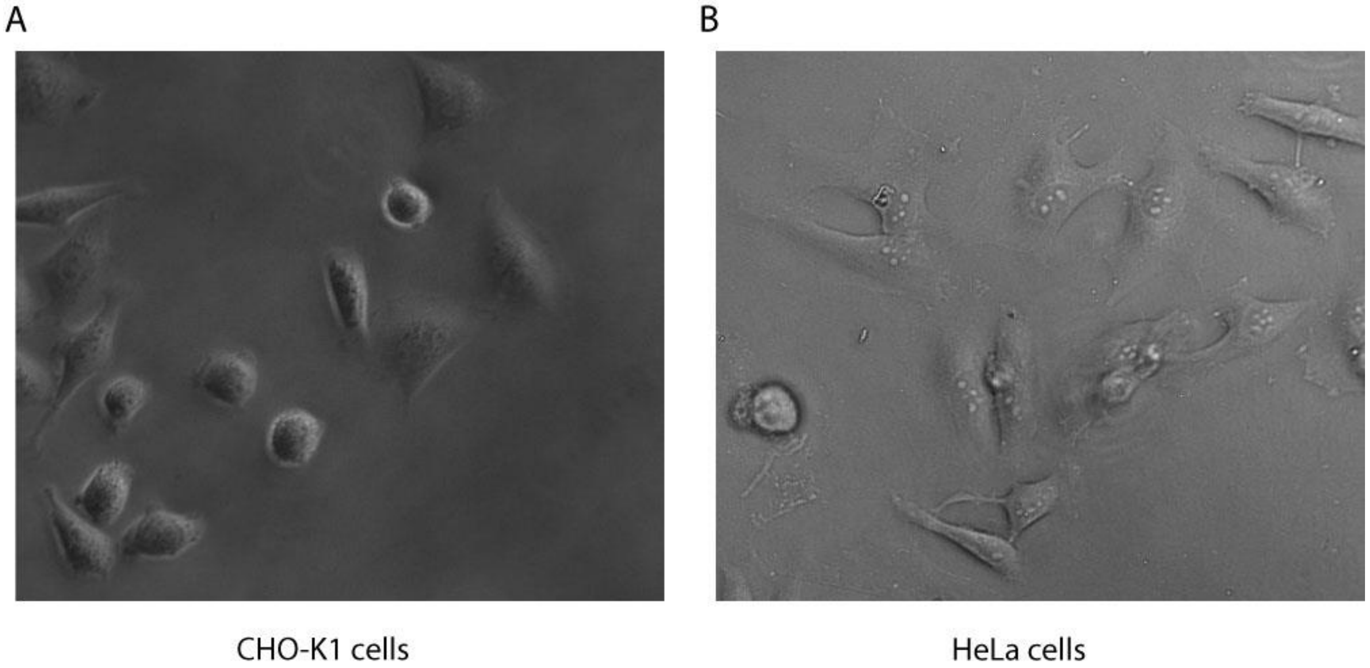

Figure 1: Example images of CHO-K1 cells imaged with Ph1 (A) and HeLa cells imaged with Ph2 (B).

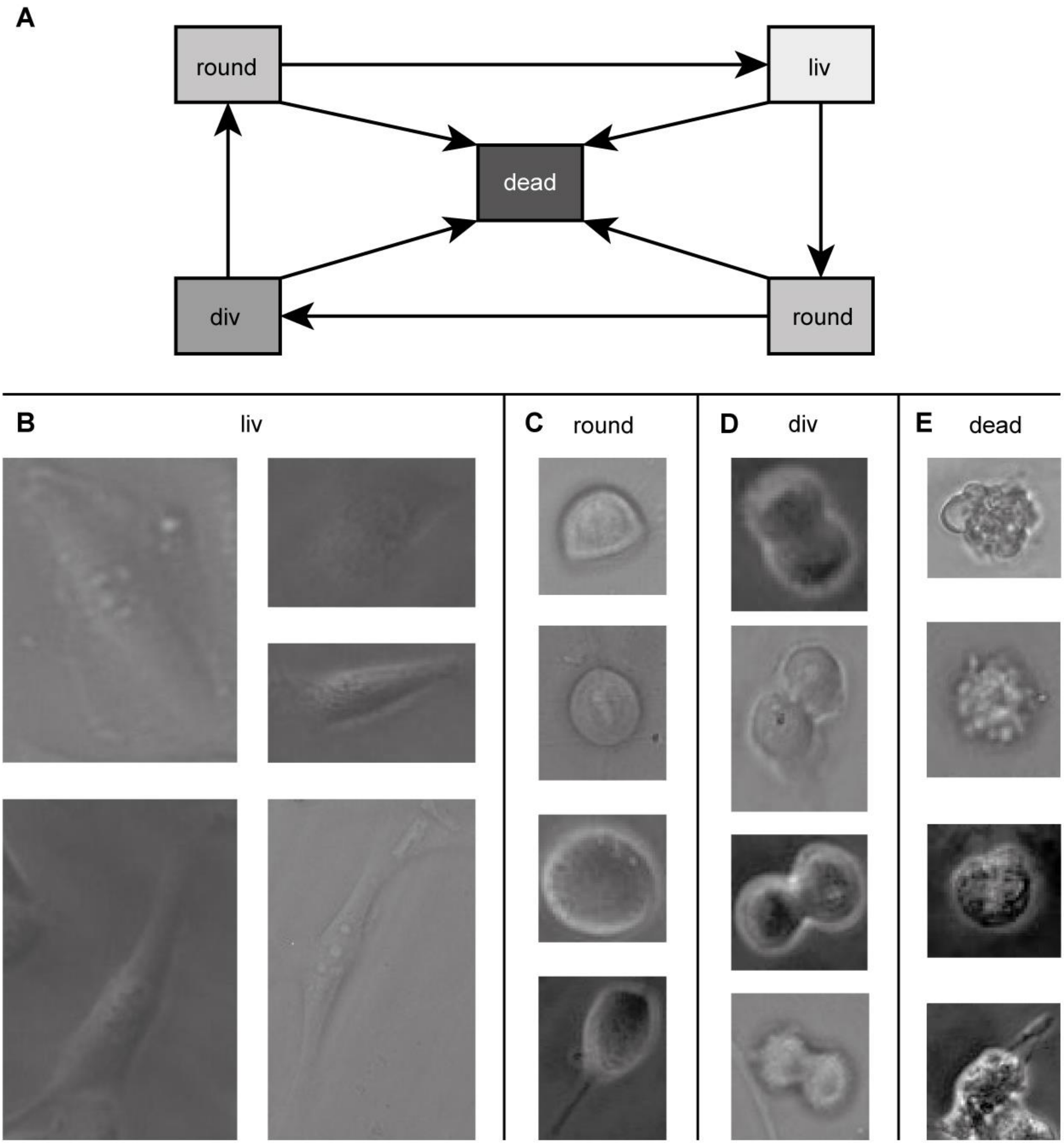

Figure 2: Figure 1 in the paper with raw data images.

Video 1: The phase-contrast video of the 4 Gy irradiated cells. The predictions from CeCILE here are shown by white boxes.

Video 2: The phase-contrast video of the sham irradiated cells. The predictions from CeCILE here are shown by white boxes.
